# Assessing Perceptions and Adaptation Responses to Climate Change among Small-Scale Fishery on the Northern Coastal of Bengkulu, Indonesia

**DOI:** 10.1155/2023/8770267

**Published:** 2023-01-17

**Authors:** Gita Mulyasari, Agung Trisusilo, Nola Windirah, Ira Nurhayati Djarot, Agusta Samodra Putra

**Affiliations:** ^1^Department of Agricultural Socio-Economics, University of Bengkulu, Jl. WR. Supratman, Kandang Limun, Bengkulu 38371, Indonesia; ^2^Postdoctoral at Research Center for Sustainable Production System and Life Cycle Assessment, National Research and Innovation Agency, PUSPIPTEK Area, Serpong 15314, Indonesia; ^3^Research Center for Sustainable Production System and Life Cycle Assessment, National Research and Innovation Agency, PUSPIPTEK Area, Serpong 15314, Indonesia

## Abstract

Small-scale fisheries are facing significant challenges from climate change. Fishers feel the impact of climate change, which forces them to adapt. We, therefore, analyzed local climatic changes, fishers' perceptions regarding climate change and its impacts, adaptation responses, and determinants. Three decades of meteorological data were analyzed (1985–2020). A total of 300 fishermen were selected using quota sampling and interviewed using a structured questionnaire. Data were analyzed using the descriptive and binary logit regression models to explain the determinants of adaptation responses. The findings indicate that fishers' perceptions of climatic changes align with historical climatic data. Typologies of adaptation responses used in the study showed that time fishing adjustment was the most widely used adaptation option by fishermen. For this reason, fishermen are very active in looking for information about climate change to help them find the right time to go to sea and reduce the risk of climate change. Analysis using the binary logit regression model showed that fishing income, boat power, and climate change perceptions were the significant (*p* < 0.1) factors significantly influencing adaptation responses. Therefore, to strengthen the adaptation responses in small-scale fisheries, fishers' perceptions should be considered.

## 1. Introduction

Climate change has a serious impact on coastal areas. Coastal areas are the areas which are most vulnerable to the adverse effects of global warming due to the accumulation of land and ocean influences. In recent years, fishers have felt a change in seasonal patterns in line with climate change due to global warming. There are two types of impacts caused by climate change. The two impacts are ecological and socioeconomic impacts. Ecological changes that occur are changes in fishing seasons, changes in fishing areas and seasons, increased risk of going to sea due to extreme waves and strong winds, and hindering fisherman's access to fishing due to the shallowing of the estuary and large waves. Changes in fish seasons are caused by an increase in ocean temperature and sea salinity, which results in the movement of fish [1].

Meanwhile, the uncertainty of fishermen's income is a socioeconomic impact of climate change. Fishermen are very dependent on natural conditions in the sea, which will determine what they catch. The presence of climate change, such as rainfall and high waves accompanied by strong winds, makes fishing less time-consuming and less profitable. According to [2, 3], another impact of climate change has an impact on changes in fishermen's production activities.

The coastal area of Bengkulu is a unique ocean because of its geographical location, which is located between the continents of Asia and Australia and is directly adjacent to the high seas, namely the Indian Ocean; this boundary directly forms the characteristics of the oceanographic parameters that occur on the west coast of Sumatra. The coastal area of Bengkulu is more subject to abrasion and has the characteristics of deeper waters because it borders the Indonesian Ocean, which has larger currents and waves. This condition causes the Bengkulu Coastal Area to be very vulnerable to climate change. The height of the waves and the location directly facing the ocean have the potential for high shoreline changes. In addition, the potential for a tsunami due to its proximity to the meeting zone of the Eurasian and Indo-Australian plates causes the Bengkulu Coastal Area to need attention, especially for coastal research related to climate change.

The adaptation highlights the importance of human beings (fishers) in executing rational, effective, and cost-efficient strategies to respond to uncertain climatic conditions. The microlevel adaptation process implies agents' interdependence through their relationships with each other, with the institutions in which they live, and with the resource base on which they depend [4]. Determining the right strategy requires fishers' knowledge and perceptions of changes in climate indicators. This perception will affect the adaptation actions that fishers will take. Fishers will experience climate change in different ways, which will be influenced by the different characteristics of the coastal area. The differences in the perceived impacts of climate change will provide perceptions and levels of adaptation that also differ from one region to another. Research in Africa shows that community adaptation to climate change differs from one region to another, even within one country [5]. Seeing the context of fishers as a community group that is vulnerable to climate change, this study aims to explore the perceptions of fishers and the adaptations carried out by fishers in dealing with climate change.

## 2. Conceptual Framework

The definition of small-scale fisher itself is stated in several laws, including Law 45/2009 on fisheries, which states that small-scale fishers are people whose livelihoods are fishing to meet the needs of daily life using fishing vessels with a maximum size of five gross tons (GT). Law 23/2014 concerning regional government defines small-scale fishers, namely traditional fishers who use traditional fishing materials and tools, as not subject to business licenses, free from taxes, and free to catch fish in all fisheries management within the territory of the Republic of Indonesia. Even the most recent rule, Law No. 7/2016 concerning the protection and empowerment of fishermen, fish cultivators, and salt farmers defines small-scale fisheries as fishers who carry out fishing activities to meet their daily needs, both those who do not use fishing vessels and those who use fishing vessels with a maximum size of ten gross tons (GT).

**Figure 1:**
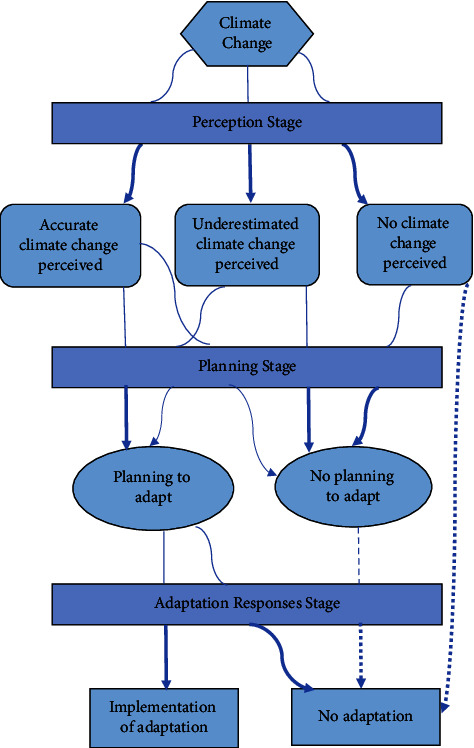
Conceptual framework of adaptation response stages.

Climate change is perceived by fishermen as a result of the following: (1) changes in temperature and rainfall [6–14]; (2) wind changes [15–17]; (3) extreme climate events such as floods and droughts [11, 15]; and (4) a shift in the seasons [18]. The impact of climate change is so large that it requires active efforts to anticipate it. Adaptation strategies for dealing with climate change are needed to reduce its impact [19]. Adaptation is an important concept in climate change, disaster risk, and the socioecological framework. Adaptation is human behavior deviating from its state in response to pressure or other driving effects. Adaptation is generally perceived as an adjustment made in response to environmental changes and their actual and expected impacts. Various researchers have put forward various definitions of adaptation, but “adjustments to change” are the key to adaptation [20]. This study looks at the importance of fishers' perceptions of climate change to build fishers' responses to adaptation (Figure 1).

## 3. Research Methodology

### 3.1. Description of the Study Area

**Figure 2:**
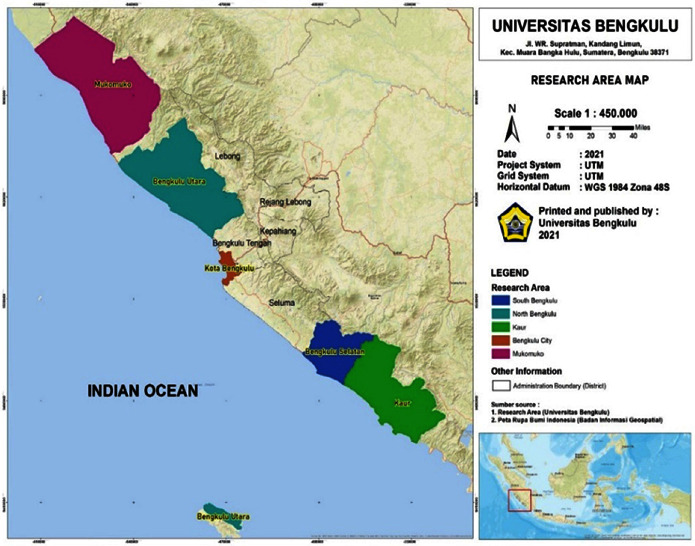
Research location processed by authors 2021.

This research was conducted in Bengkulu Province's coastline area, which includes Bengkulu City, North Bengkulu Regency, Mukomuko Regency, South Bengkulu Regency, and Kaur Regency (Figure 2). Bengkulu Province is located in the west of the Bukit Barisan mountains. It covers an area of approximately 1,991,933 ha (or 19,919.33 km^2^) and extends from the border of West Sumatra Province to the border of Lampung Province, the distance is approximately 567 km.

Bengkulu Province is located between 2°16′ and 3°31′ south latitude and between 101°01′ and 103°41′ east longitude. Bengkulu Province has a coastline of approximately 525 km that is directly adjacent to the Indonesian Ocean (Indian Ocean). The eastern part is hilly with fertile highlands, while the western part is a relatively narrow lowland, extending from north to south and interspersed with undulating areas. Bengkulu Province is considered to be the center of the world's climate, where the waters in Bengkulu Province become a meeting place for four ocean currents, eventually becoming an area where the process of evaporation of rain cloud formation occurs, resulting in the rainy or dry season and affecting the world's climate [21].

### 3.2. Sampling and Data Collection

**Figure 3:**
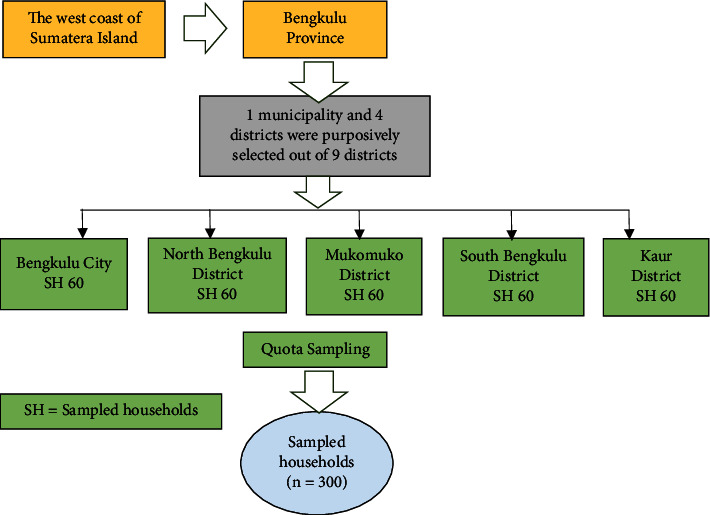
Schematic presentation of the sampling procedure.

In this study, respondents were chosen from the study area using a multistage sampling technique. Bengkulu Province is one of Sumatra Island's western coastal areas that was purposely chosen as the research area due to its high vulnerability to climate change. In the second stage, one municipality and four districts were purposefully selected from the district's total. In the third stage, quota sampling for each research location is required since the number of small coastal fisher households in each research location is not known with certainty (Figure 3).

Data for this study were collected through a questionnaire survey of small-scale fishery households in the coastal Bengkulu Province. The purposive sampling method was employed to determine the respondents. Small-scale fisheries in this study are artisanal or traditional fisheries that involve fishing households, use relatively small fishing vessels (≤10 GT), short fishing trips (one-day fishing), and use relatively small capital and manpower. Before conducting the survey, it is necessary to assess the validity and reliability of the research instrument to ensure the validity and consistency of the question items in the questionnaire, particularly addressing the qualitative variables of fishers' perceptions of climate change. This study's validity and reliability tests were carried out for climate change perceptions, and climate change impact perceptions (see Tables 1 and 2). The questionnaire was divided into four main parts: the first part collected respondents' demographic information, including their age, education level, fishing experience, household size, income, and side jobs. The second part collected the characteristics of the small-scale fishery, including boat power, fishing days, fishing time, fishing distance, catch capacity, fishers' group membership, access to climate information, and access to credit.

The third part of the questionnaire focuses on fishermen's perceptions of perceived changes in climatic indicators, as well as the impact of climate change on capture fisheries businesses. Climate change is assessed based on small-scale fisheries' perceptions of changes in 10 climate indicators based on previous research [3, 7, 22–26] as measured by Likert, with a score of 1 if it decreases, a score of 3 if it remains, and a score of 5 if it increases. The impact of climate change on capture fisheries businesses as viewed by small-scale fishermen was also assessed by asking climate change-related impact questions [3, 18, 22, 27–38].

The last part of the questionnaire is the response to fishers' adaptations to climate change. Based on previous research [39–43], this study employs 16 adaptations that can assist fishermen in increasing their adaptive capacity in response to the impacts of climate change. The adaptation response of fishermen is categorized as high (score 1) if the number of adaptations carried out is 50% of the indicator and low (score 0) if the number of adaptations carried out by fishermen in reducing the impact of climate change is less than 50% of the indicator.

### 3.3. Data Analysis

A binary logit model was used to analyze the factors determining fishers' adaptation responses since this model is frequently used in similar previous studies [44–47], whether a household adopted a high adaptation measure (1) or a low adaptation measure (0). Empirical studies [46, 48–56] show that households' adaptation responses are shaped by various socioeconomic characteristics, fishery-related attributes, and their access to various institutional services. A detailed description of these independent explanatory variables is given in Table 3.

**Figure 4:**
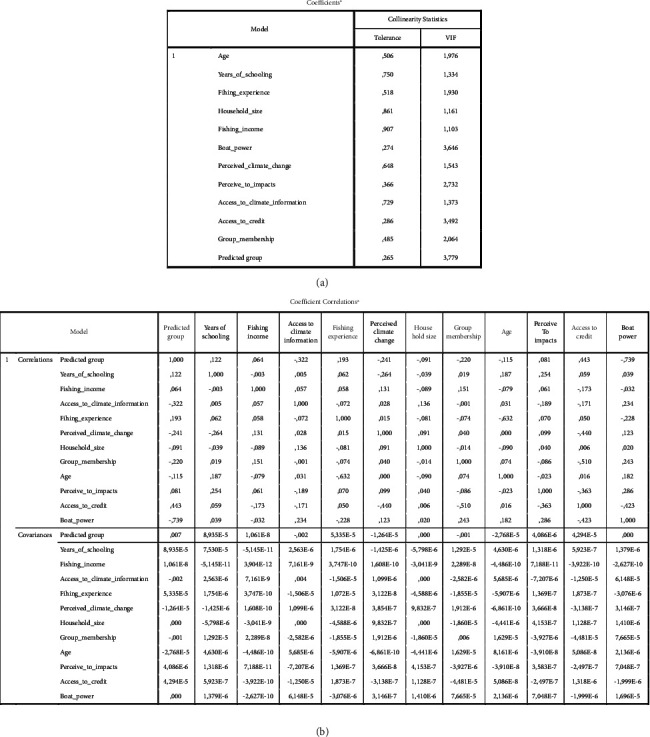
The result of multicollinearity.

Before estimating the binary logit model, it was needed to determine whether the selected explanatory variables were associated or correlated with each other. This was done using the variance inflation factor (VIF) and contingency coefficient (CC) to check the multicollinearity effect or any associations between continuous explanatory variables (Figure 4). According to [57], the existence of multicollinearity may seriously affect parameter estimates of the logit model.

## 4. Characteristics of Small-Scale Fishery

### 4.1. Socioeconomic Characteristics

**Figure 5:**
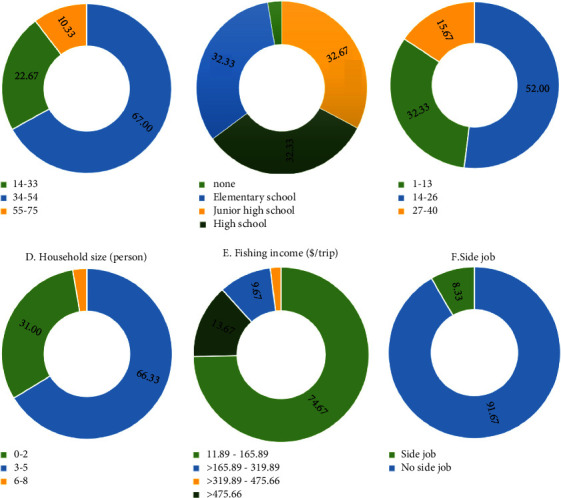
Percentage of the respondents based on sociodemographic characteristics (source: field survey, 2021): (a) age (years), (b) education (years), (c) experience (years), (d) household size (person), (e) fishing income ($/trip), and (f) side job.

The socioeconomic characteristics of the respondents and the household including age, education, fishing experience, household size, fishing income, and a side job (Figure 5).

### 4.2. Small-Scale Fishery Characteristics

Attributes characterizing the study participants, including boat power, fishing days, fishing time, fishing distance, catch capacity, group membership, access to climate information, and access to credit, were collected using multiple-choice responses and are presented in Table 4.

## 5. Results and Discussion

### 5.1. Meteorological Analysis

**Figure 6:**
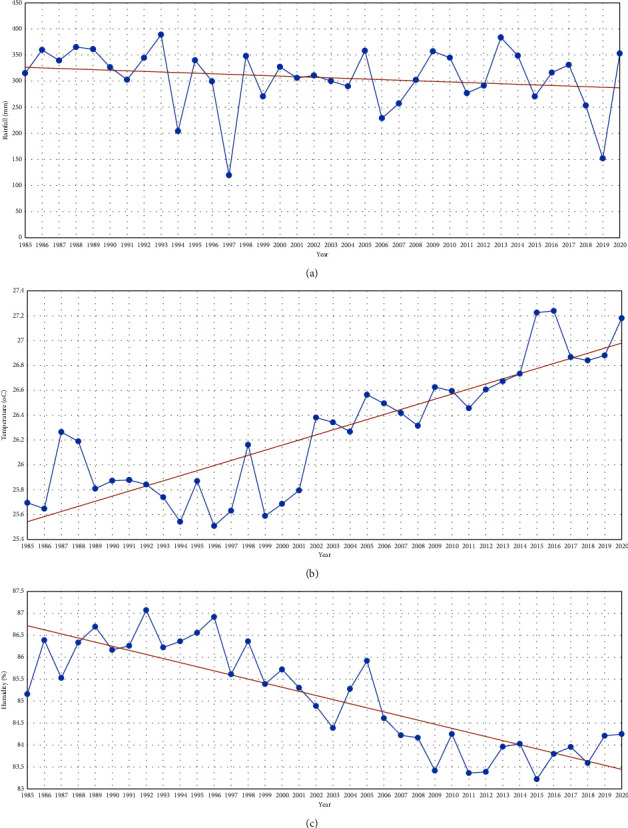
Graphs showing (a) average annual amount of rainfall trends, (b) average annual temperature trends, and (c) average annual humidity trends in Bengkulu Province (1985–2020) (source: BMKG Bengkulu Province, 2021).

The coastal area of Bengkulu has very variable rainfall (Figure 6(a)), depending on the topography. Rainfall varies from >6000 mm per year in the western area of Bukit Barisan to less than 1500 mm per year in the eastern area of Bukit Barisan where Bukit Barisan and the Malay Peninsula block moisten air. However, in general, the station recorded that 70% of the Bengkulu area received more than 2500 mm of rainfall per year. [58] explains a downward trend in annual rainfall in Bengkulu Province with a value of 71.79 mm/year from 1968 to 1997. The dry season in most of the Bengkulu area is related to the northeast monsoon, which occurs between December and March, while the main rainy season occurs during the transition period before the northeast monsoon and after the southwest monsoon, which lasts from May to September. The secondary rainy season occurs around April.

Climate change, such as changes in wind conditions, erratic rains, and the difficulty of determining the location of fish catches due to changes in currents, causes fish to be unable to stay in one place for a long time. For fishers in the western coastal area of Bengkulu, directly facing the Indonesian Ocean, this changing season pattern is accompanied by high waves and strong winds that can sink fishing boats. Many fishermen miscalculate the pattern of the season when leaving for the sea. The impact can endanger the safety and catches of fishermen. Based on Figure 6(a), it can be seen that there was a decrease in rainfall, which was quite extreme in 1997. According to [59], 1997 was a dry year because the El Nino phenomenon took place, which was also the year of the Indian Ocean Dipole Mode (IODM) in the Indian Ocean. In general, in Indonesia in 1997, there was an extraordinary drought due to the rainy season, which occurred in a short period. In addition, there were numerous forest and bushfires in Indonesia in 1997, particularly on Sumatra.

Figure 6(b) shows a significant increase in the average annual temperature in the Bengkulu coastal area (Table 5), ranging from 1.1°C to 1.2°C. Areas with relatively warmer sea surface temperatures are in the waters west of Bengkulu, where there are temperature anomalies, the sea level reaches +1°C [60]. Facts show that the average air temperature in 1850 differed significantly from the current situation. From 1850 to 1910, there was a climate anomaly with the rise and fall of the average temperature of the Earth's surface. The temperature in the Bengkulu Coastal Area also varies, but the annual fluctuation is very small, and the temperature difference is more influenced by the altitude.

Overall, the humidity in the Bengkulu Coastal Area decreased (Table 5 and Figure 6(c)) along with the increased air temperature. Climate change greatly affects individual's, population's, and the community's physiology and behavior. Fish can die in extreme conditions such as increased water temperature, low dissolved oxygen concentration, and low water pH. Environmental conditions that are not optimal can reduce the metabolic rate, growth, and egg-laying ability of fish, change metamorphosis, and affect the endocrine system and crocodile patterns [28]. These changes directly affect the fish population and community structure, which in turn affect the fishery stock.

Fishermen also have local knowledge that can be used to deal with conditions at sea. However, some of the local knowledge that fishers currently have is not always appropriate in uncertain conditions. So, local knowledge needs to be balanced with global knowledge, namely, knowledge about climate change [61, 62]. In the Bengkulu Coastal Area, fishers understand very well that there is an increase in extreme weather events due to the coast's characteristics that are directly opposite to the Indian Ocean, which is prone to high waves, storms, and strong winds, for example, the Durga Cyclone in the waters southwest of Bengkulu (April 22–25, 2008), the Orchid Cyclone in the waters west of Bengkulu (October 30–November 4, 2010), and the Daffodil Cyclone in the waters southwest of Sumatra (December 11–13, 2014).

The west coast of Bengkulu is more subject to abrasion. It has the characteristics of deeper waters because it borders the Indonesian Ocean, which has larger currents and waves, so that fishers can understand various changes in climate indicators. Extreme events such as high waves, storms, and big waves are more common on the Bengkulu Coast, so fishers understand this indicator well (Table 6).

Fishermen, for example, are familiar with sea currents, which, according to some sources, typically occur between the 15th and 21st of every month. Outside of these dates, the ocean currents are usually calm. If there is a sudden change, fishermen can feel it through the rising waves. Fishermen on the coast of Bengkulu can also know that the wind that resembles a cloud that is seen hanging above the sea surface is a sign of strong winds. The fishermen finally did not dare go to sea in such conditions.

The occurrence of weather anomalies causes fishers to have difficulty predicting the start of the west season (wave season) and east season (shady season), whereas certainty is very important because fishermen use small boats, they are very sensitive to changes in weather. Not infrequently, due to sudden weather changes, they are forced to return to land because they see a cloud hanging in the middle of the sea, which is considered a sign of strong winds. The west monsoon has also become longer than usual as a result of climate change. Climate change not only makes the lives of fishermen more uncertain, but it also makes their lives harder.

### 5.2. Adaptation Responses

Several responses are used for adaptation to climate change in small-scale fisheries, e.g., diversification of fishing gears, timing fishing adjustments, utilization of social networks, and changes in the fishing grounds. The adverse effects of climate change, such as sea-level rise, coastal erosion, extreme waves, and tidal flooding, have significantly reduced fishing productivity. According to [63, 64], these effects hurt small-scale fisheries the most because they mostly depend on capture fisheries.

Our interviews revealed that fishers with experience of climatic stresses were more likely to implement adaptation strategies, upholding the theory that risk experience influences response behavior [65, 66]. Fishers implemented a wide range of adaptation strategies, but the most commonly mentioned strategies were to adjust the fishing time and look for information about weather and climate change, with reduced climate vulnerability as a secondary benefit (Table 7).

For example, climate change that causes extreme waves and strong winds often occur in the east monsoon season and during peak fishing activities. This is certainly very detrimental, where boats and fishing facilities on the coast of Bengkulu are still traditional and not yet equipped to face storms or big waves. So fishers need to adjust their fishing and fishing activities to reduce the risk of climate change.

The majority of fishers in Bengkulu also choose to look for complete information about weather and climate change, with the use of social networks as the most widely used adaptation option to reduce the impact of climate change (Table 7). Fishermen often access weather information from the BMKG website as a reference for going to sea. On the coast of Bengkulu, fishers have limited economic and financial resources, so the adaptation option is the most widely used because it does not require a lot of economic resources, technology, or other resources beyond the capabilities of fishers. Climate change causes changes in sea surface temperature and water column stratification, affecting the upwelling of the ocean [67]. Changes in ocean circulation and the upwelling process cause changes in fish migration patterns and fish schools [67]. This is also in line with the opinion of an ecologist [68], who states that the most productive part of the ocean is where the upwelling occurs. Fish migration, which occurs when schools of certain fish cross a fishing area, is what fishermen refer to fishing season or fish migration. This change in fish seasons greatly affects fishers' income, considering that some fish species only come in certain seasons. According to fishers, climate change is thought to have significantly impacted the fishing season.

Poverty in fishing communities causes fishers to have limited resources, especially economic resources. Another adaptation option that is mostly used by fishermen is the diversification of fishing gear. Most fishers use fishing nets as their main fishing gear because they are easy to use and safe for coastal ecosystems. Fishers use fishing rods or strings as additional fishing gear in addition to nets because they are inexpensive and simple to use. So that when fishers spread their nets and wait for the nets to be pulled, they can catch more fish.

Binary logistic regression has been used to identify factors determining fishers' responses to climate change effects. The response of fishers is a discrete value (1, 0). One (1) denotes fishers who implement more than 50% of the adaptation strategies in this study to climate change, while zero (0) denotes fishers who adapt to climate change with less than 50% of the adaptation strategies in this study (the data for binary logistic regression can be seen in the supplementary file).

Boat power (coefficient = 6.51, *p* < 0.01) and perceived climate changes (coefficient = 2.12, *p* < 0.05) both had a positive relationship with the adaptation response (Table 8). The result is conceivable because ships with greater power are better able to deal with changes in climate indicators than small boat fishermen. Strong typhoons or severe weather can also destroy fishing gear such as boats and nets, making it necessary to be prepared for repair or replacement [69]. We have noted during our interviews that the lack of financial support was mentioned by the fishers as a problem for boat and gear replacement, boat repairs, starting a new business, or emergency loans [70, 71]. In addition, the perception of climate change received by fishers influences their readiness to adapt to various impacts caused by climate change. The significant relationship between these variables and adaptation response is consistent with previous studies [50, 72].

Fishermen in Bengkulu are small-scale fishers who only use small fishing fleets, so they are very vulnerable to the impacts of climate change. The study [73] explains that to improve the conditions of fishermen due to climate change, an effort is needed to change their fishing operation strategy, among others, by increasing the size of the boat, moving fishing grounds, and extending fishing operation time during the harvest season.

In Indonesia, a strategy has never been implemented for fishing communities to increase the weight of their vessels. The government, through the Ministry of Maritime Affairs and Fisheries (KKP), built 994 government-assisted fishing vessels in 2017. Of these, 449 units of ships will be built under 5 GT, 384 units of 5 GT ships, 134 units of 10 GT ships, 15 units of 20 GT ships, 6 units of 30 GT ships, and 3 units of 120 GT ships. In addition, 3 transport ships with a freezer capacity of 100 GT will also be built. This fishing vessel assistance is intended for local fishermen so that they can optimally and sustainably take advantage of the increasingly abundant fish resource stock. In addition, it is important to increase the added value and competitiveness of fishery products because it can increase the income and welfare of fishermen.

Fishermen in Bengkulu have a low understanding of climate change. Fishers only understand the changes in the west monsoon and east monsoon winds, which are the references for the fisher to go to sea and catch fish. The study [74] also reveals that only a small number of fisher understand the increase in temperature as a result of global warming. Fishers' understanding of climate change is more influenced by the El Nino and La Nina phenomena that occur and can be felt significantly by fishermen. In addition, fishermen are more familiar with the term weather due to the lack of information that they get and their low level of education. With this low level of understanding, the level of adaptation of fishers to climate change will also be low.

The author in [75] states that climate change has changed the fisher's knowledge system about natural conditions. Their knowledge that has been adapted to natural conditions in the past is considered no longer able to adapt to current climate changes. Furthermore, the authors [76] explained that the understanding of traditional fishing communities about climate change is based on experience and not on the latest climate change science. Due to the difficulty of determining fishing areas, fishermen have to spend more money to go to sea because they have to move from one fishing ground to another. In addition, fishers also have to increase the number of fishing days from once a day to several times a day because of the erratic weather.

Overall, variables such as age, years of schooling, fishing experience, household size, fishing income, boat power, perceived climate change, perceived climate impacts, access to climate information, access to credit, and group membership simultaneously (Prob > Chi^2^, *p* < 0.01) had significant effects on the adaptation response to climate change with a determinant coefficient of 45.7% (Table 8).

## 6. Conclusions and Policy Implications

The findings of this study are that fishers consistently realize that there has been a change in climate indicators, which is in line with the historical data on climate change (1985–2020) analyzed in this study. For example, fishers realize that there has been an increase in temperature in the Bengkulu Coastal Area. This finding is in line with data on temperature changes, which also show a significant increase. This also applies to data on changes in other climate indicators that align with how fishermen feel about climate change and its impacts. Another finding in this study is that fishermen with more experience prefer to adapt. Climate change is expected to have a tremendous impact on fishermen's livelihoods. Fishermen must adapt to their fishing and fishing activities to reduce the risks from climate change. From our analysis using the binary logit regression model, surprisingly, the coefficient of fishing income was negative and significantly affected the adaptation response, but the fishers' perception of climate change significantly determined the adaptation responses. Therefore, to strengthen the adaptation responses in small-scale fisheries, fishers' perceptions should be considered.

Climate change adaptation activities in Indonesia must be carried out in an integrated manner with development programs, particularly in the fisheries sector. Programs and adaptation actions that are developed and implemented in Indonesia must involve fishers with regard to the risks and threats that are being felt by fishers now and in the future. Immediate adaptation programs and actions are directed at coastal areas that do have a high level of climate risk and are expected to remain high or tend to increase in the future.

## Figures and Tables

**Table 1:** Validity and reliability results of climate change perceptions.

Perceived changes	Corrected item-total correlation	*r* table	Result
Annual temperature	0.641	0.378	Valid
Intensity of rainfall	0.929	0.378	Valid
Frequency of rainy days	0.929	0.378	Valid
Sea-level rise	0.832	0.378	Valid
Direction and wind speed	0.700	0.378	Valid
Direction and strength of ocean currents	0.770	0.378	Valid
Wave height	0.700	0.378	Valid
The intensity of extreme natural events	0.852	0.378	Valid
Wave strength	0.454	0.378	Valid
Average wave height	0.712	0.378	Valid
Extreme temperature	0.626	0.378	Valid
Cronbach's alpha based on standardized items = 0.905			

*r* table = DF_*N* − 2_;*α* = 20-2; 0.05 = 18; 0.05 = 0.378. Software SPSS 23.

**Table 2:** Validity and reliability results of climate change impact perceptions.

Perceived fisheries impacts	Corrected item-total correlation	*r* table	Result
Changes of the east monsoon and west monsoon	0.545	0.378	Valid
Difficult to predict the fishing time	0.761	0.378	Valid
Difficult to determine the wind direction	0.790	0.378	Valid
Difficult to predict the coming of a storm	0.821	0.378	Valid
Changes in fish distribution/fish migration	0.762	0.378	Valid
Decreasing fish population	0.733	0.378	Valid
Decreasing fish production	0.543	0.378	Valid
Decreasing fish species catch	0.698	0.378	Valid
Potential catch reduction	0.734	0.378	Valid
Changes in the reproduction pattern of caught fish	0.677	0.378	Valid
Coral bleaching	0.794	0.378	Valid
Decreased area of mangrove forests, estuaries, and swamps	0.736	0.378	Valid
Changes in fishing seasons	0.592	0.378	Valid
Changes in fish breeding habitats	0.581	0.378	Valid
Difficult to determine fishing areas	0.737	0.378	Valid
Higher levels of abrasion and tidal waves	0.690	0.378	Valid
Higher risk of fishing activities	0.590	0.378	Valid
Reduced availability of clean water	0.688	0.378	Valid
Seawater intrusion	0.763	0.378	Valid
Increased operating costs at sea	0.704	0.378	Valid
Decrease in the income of fishers	0.762	0.378	Valid
Unfulfilled conditions of food security	0.680	0.378	Valid
Cronbach's alpha based on standardized items = 0.957			

*r* table = DF_*N* − 2_;*α* = 20-2; 0.05 = 18; 0.05 = 0.378. Software SPSS 23.

**Table 3:** Description of the variables.

Variables	Measurement	Expected sign	Mean	S.D
Age	The number of years from birth	+/−	41.05	10.50
Years of schooling	Total number of years of formal education	+/−	8.77	2.84
Fishing experience	Total number of years into fishing	+/−	17.51	9.06
Household size	Size of respondent's household	+/−	3.02	1.32
Fishing income	Monthly fishing income in dollars	+	303.09	583.32
Boat power	Engine size of the boat (horsepower)	+	24.93	10.02
Perception of climate change	Score	+	38.94	4.28
Perception of climatic impacts	Score	+	75.85	12.96
Access to climate information	Dummy (1 = fishers can access climate information, 0 = otherwise)	+	0.19	0.39
Access to credit	Dummy (1 = fishers can access credit, 0 = otherwise)	+	0.14	0.35
Fishers' group membership	Dummy (1 if a fisher belongs to a fishing group and 0 if not)	+	0.20	0.40

**Table 4:** Small-scale fishery characteristics (*n* = 300).

Characteristics	Category	Frequency	Proportion (%)
Boat power (horsepower)	3.5–15	125	41.67
	16–27	64	21.33
	28–40	111	37.00
Fishing days (days/month)	≤10	2	0.67
	11–20	256	85.33
	21–30	42	14.00
Fishing time (hours)	3–5	57	19.00
	6–8	181	60.33
	9–12	62	20.67
Fishing distance (miles)	1–5	137	45.67
	6–10	78	26.00
	11–15	18	6.00
	>15	67	22.33
Catch capacity (kg/trip)	<100	192	64.00
	100–200	76	25.33
	>200–300	8	2.67
	>300	24	8.00
Fishers' group membership	Yes	61	20.33
	No	239	79.67
Access to climate information	Yes	57	19.00
	No	243	81.00
Access to credit	Yes	46	15.33
	No	254	84.67

Source: Field survey, 2021.

**Table 5:** Results of Mann–Kendall analysis for selected climatic variables.

Variables	Mean	Std. deviation	Sig.	Trend
Rainfall	306.857	59.558	0.145^ns^	No trend
Temperature	26.261	0.510	0.0001^*∗∗∗*^	Upward trend
Humidity	85.082	1.175	0.0001^*∗∗∗*^	Downward trend

Source: BMKG Provinsi Bengkulu, 2021. ^ns^: not significant. ^*∗∗∗*^significant at 1%.

**Table 6:** Perceived changes in the climate and within the fisheries (*n* = 300).

*Perceived changes*	*Decrease*	*No change*	*Increase*

Annual temperature	8.33	9.00	82.67
Amount of rainfall	80.33	17.33	2.33
Frequency of rainy days	2.33	13.00	84.67
Sea-level rise	0.00	27.00	73.00
Direction and wind speed	0.00	26.67	73.33
Direction and strength of ocean currents	0.00	21.33	78.67
Wave height	0.00	19.33	80.67
The intensity of extreme natural events	0.00	14.00	86.00
Wave strength	4.00	16.00	80.00
Average wave height	27.67	25.67	46.67
Extreme temperature	20.67	10.67	68.67

*Perceived fishery impacts*	*Disagree*	*No impact*	*Agree*

Changes of the east monsoon and west monsoon	9.33	39.33	51.33
Difficult to predict the fishing time	24.33	22.67	53.00
Difficult to determine the wind direction	33.00	30.00	37.00
Difficult to predict the coming of a storm	19.00	32.33	48.67
Changes in fish distribution/fish migration	14.00	50.67	35.33
Decreasing fish population	20.00	30.00	50.00
Decreasing fish production	23.00	36.67	40.33
Decreasing fish species catch	15.00	50.33	34.67
Potential catch reduction	19.00	42.33	38.67
Changes in the reproduction pattern of caught fish	16.33	46.00	37.67
Coral bleaching	35.67	34.33	30.00
Decreased area of mangrove forests, estuaries, and swamps	15.33	41.00	43.67
Changes in fishing seasons	2.67	45.67	51.67
Changes in fish breeding habitats	17.33	49.33	33.33
Difficult to determine fishing areas	34.67	38.33	27.00
Higher levels of abrasion and tidal waves	9.00	35.67	55.33
Higher risk of fishing activity	0.67	13.33	86.00
Reduced availability of clean water	23.33	53.00	23.67
Seawater intrusion	0.67	52.33	47.00
Increased operating costs at sea	0.67	59.33	40.00
Decrease in the income of fishers	10.00	43.33	46.67
Unfulfilled conditions of food security	17.33	58.00	24.67

Source: field survey, 2021.

**Table 7:** Adaptation responses by small-scale fishery households (*n* = 300).

Adaptation responses	Yes (%)
Adopted wanamina (a combination of mangrove forest and fish farming)	12.00
Strengthening fishers' institutions for resilience to climate change	27.33
Planting mangroves or other coastal vegetation in coastal areas	28.00
Making APO (breakwater)	29.67
Increase knowledge and information about climate change through Climate Field Schools, early warning systems, and climate information network systems	35.67
Diversification of economic activities	40.33
Develop boats that are resistant to weather changes and big waves	43.67
Mobilization of family members to work	45.00
Diversification of fishing gears	50.33
Build fishing settlements designed to anticipate sea-level rise	56.00
Changes in the fishing ground	67.00
Use of geoinformation and communication technology systems such as GPS and fishfinder	74.33
Make adjustments or replace the fishing gear used	81.33
Utilization of social networks	83.00
Looking for information about weather and climate change	87.00
Time fishing adjustment	90.33

Source: Field survey, 2021.

**Table 8:** Results of binary logistic regression of adaptation responses.

Variables	Odds ratio	Std. error	*z*	*P* > |*z*|
Cons	0.005	0.015	−1.87	0.062
Age	1.001	0.021	0.04	0.970^ns^
Years of schooling	0.995	0.066	−0.08	0.939^ns^
Fishing experience	0.961	0.024	−1.54	0.123^ns^
Household size	1.088	0.146	0.63	0.529^ns^
Fishing income	0.998	0.001	−1.95	0.051^*∗*^
Boat power	1.229	0.039	6.51	0.000^*∗∗∗*^
Perceived climate changes	2.834	1.392	2.12	0.034^*∗∗*^
Perceived climate impacts	0.609	0.251	−1.20	0.229^ns^
Access to climate information	1.272	0.583	0.53	0.599^ns^
Access to credit	0.428	0.482	−0.75	0.452^ns^
Group membership	0.005	0.015	1.20	0.228^ns^
Number of obs = 300				
Log likelihood = −106.054				
LR Chi^2^ (11) = 178.78				
Prob > Chi^2^ = 0.0000^*∗∗∗*^				
Pseudo *R*^2^ = 0.457				

Source: field survey, 2021. Software: STATA version 15. ^ns^: not significant. ^*∗∗∗*^significant at 1%. ^*∗∗*^significant at 5%. ^*∗∗*^significant at 10%.

## Data Availability

The data used to support the findings of this study are available from the corresponding author upon request (Gita Mulyasari—gita.mulyasari@unib.ac.id).

## Supplementary Materials

Supplementary Materials This article has figures to clarify information consisting of (1) a conceptual framework which describes the theoretical framework regarding the stages of implementing climate change adaptation; (2) a map of research locations explaining where this research was carried out specifically and visually; (3) taking and determining respondents using the multistage sampling technique; (4) socioeconomic characteristics of respondents including age, education, experience, household size, fishing income, and side job; and (5) trend indicators of climate change including annual rainfall, temperature, and humidity from 1985 to 2020.

## References

[1] Patriana R. (2011). Fishermen adaptation patterns to climate change (case study of fishermen in ciawitali hamlet, pamotan village, kalipucang district, ciamis regency, west java).

[2] Chen J., Yin S., Gebhardt H., Yang X. (2018). Farmers’ livelihood adaptation to environmental change in an arid region: a case study of the Minqin Oasis, northwestern China. *Ecological Indicators*.

[3] McMullen C. P., Jabbour J., Unep (2009). Climate change science compendium. http://www.unep.org/pdf/ccScienceCompendium2009/.

[4] Bazzani G. (2022). Agency as conversion process. *Theory and Society*.

[5] Nyiwul L. (2021). Climate change adaptation and inequality in Africa: case of water, energy and food insecurity. *Journal of Cleaner Production*.

[6] Bewket W. (2012). Climate change perceptions and adaptive responses of smallholder farmers in central highlands of Ethiopia. *International Journal of Environmental Studies*.

[7] Fosu-Mensah B. Y., Vlek P. L. G., MacCarthy D. S. (2012). Farmer’s perception and adaptation to climate change: a case study of Sekyedumase district in Ghana. *Environment, Development and Sustainability*.

[8] Bryant C. R., Smit B., Brklacich M. (2000). Adaptation in Canadian agriculture to climatic variability and change. *Climatic Change*.

[9] Gebrehiwot T., van der Veen A. (2013). Farm level adaptation to climate change: the case of farmer’s in the Ethiopian Highlands. *Environmental Management*.

[10] Kassie B. T., Hengsdijk H., Rotter R., Kahiluoto H., Asseng S., Van Ittersum M. (2013). Adapting to climate variability and change: experiences from cereal-based farming in the central rift and kobo valleys, Ethiopia. *Environmental Management*.

[11] Abdur Rashid Sarker M., Alam K., Gow J., Sarker A. R., Alam K., Gow J. (2013). Assessing the determinants of rice farmers’ adaptation strategies to climate change in Bangladesh. *International Journal of Climate Change Strategies and Management*.

[12] Roco L., Engler A., Bravo-ureta B. E., Jara-Rojas R. (2015). Farmers’ perception of climate change in mediterranean Chile. *Regional Environmental Change*.

[13] Limantol A. M., Keith B. E., Azabre B. A., Lennartz B. (2016). Farmers’ perception and adaptation practice to climate variability and change: a case study of the Vea catchment in Ghana. *SpringerPlus*.

[14] Arunrat N., Wang C., Pumijumnong N., Sereenonchai S., Cai W. (2017). Farmers’ intention and decision to adapt to climate change: a case study in the Yom and Nan basins, Phichit province of Thailand. *Journal of Cleaner Production*.

[15] Esham M., Garforth C. (2013). Agricultural adaptation to climate change: insights from a farming community in Sri Lanka. *Mitigation and Adaptation Strategies for Global Change*.

[16] Yaro J. A. (2013). The perception of and adaptation to climate variability/change in Ghana by small-scale and commercial farmers. *Regional Environmental Change*.

[17] Barrucand M. G., Giraldo Vieira C., Canziani P. O. (2017). Climate change and its impacts: perception and adaptation in rural areas of Manizales, Colombia. *Climate and Development*.

[18] Kurniawati F. (2012). Knowledge and adaptation of vegetable farmers to climate change.

[19] Surmaini E., Runtunuwu E. (2011). Efforts by the agricultural sector in dealing with climate change. *Jurnal Litbang Pertanian*.

[20] Lei Y., Wang J., Yue Y. (2014). Rethinking the relationships of vulnerability, resilience, and adaptation from a disaster risk perspective. *Natural Hazards*.

[21] Bppt (2016). Bengkulu pusat iklim dunia, agency for the assessment and application of technology Indonesia (BPPT). https://www.climate4life.info.

[22] Pachauri R. K., Reisinger A., Ipcc (2007). *Climate Change 2007: Synthesis Report. Contribution of Working Groups I, II and III to the Fourth Assessment Report of the Intergovernmental Panel on Climate Change, Core Writing Team*.

[23] Chukwukere A. O., Udodirim U., Okezie R., Sulaiman J. (2011). Climate variability and change: perceptions and adaptations in subsistence agriculture. *Indian Journal of Agricultural Research*.

[24] Ogalleh S. A., Vogl C. R., Eitzinger J., Hauser M. (2012). Local perceptions and responses to climate change and variability: the case of Laikipia District, Kenya. *Sustainability*.

[25] Dang H. L., Li E., Nuberg I., Bruwer J. (2014). Understanding farmers’ adaptation intention to climate change: a structural equation modelling study in the Mekong Delta, Vietnam. *Environmental Science and Policy*.

[26] Banerjee R. R. (2015). Farmers’ perception of climate change, impact and adaptation strategies: a case study of four villages in the semi-arid regions of India. *Natural Hazards*.

[27] Ankrah J. (2018). Climate change impacts and coastal livelihoods; an analysis of Fishers of coastal Winneba, Ghana. *Ocean and Coastal Management*.

[28] Lincoln S., Andrews B., Birchenough S. N. R. (2022). Marine litter and climate change: inextricably connected threats to the world’s oceans. *Science of the Total Environment*.

[29] Moullec F., Barrier N., Drira S. (2022). Using species distribution models only may underestimate climate change impacts on future marine biodiversity. *Ecological Modelling*.

[30] Peck M. A., Reglero P., Takahashi M., Catalan I. A. (2013). Life cycle ecophysiology of small pelagic fish and climate-driven changes in populations. *Progress in Oceanography*.

[31] Cheung W. W. L., Lam V. W. Y., Sarmiento J. L. (2010). Large-scale redistribution of maximum fisheries catch potential in the global ocean under climate change. *Global Change Biology*.

[32] Ji R., Edwards M., Mackas D. L., Runge J. A., Thomas A. C (2010). Marine plankton phenology and life history in a changing climate: current research and future directions. *Journal of Plankton Research*.

[33] Uy N., Takeuchi Y., Shaw R. (2011). Local adaptation for livelihood resilience in Albay, Philippines. *Environmental Hazards*.

[34] Sumaila U. R., Cheung W. W. L., Lam V. W. Y., Pauly D., Herrick S. (2011). Climate change impacts on the biophysics and economics of world fisheries. *Nature Climate Change*.

[35] Stocker T. F., Qin D., Plattner G.-K., IPCC (2013). Climate change 2013: the physical science basis. *Contribution of Working Group I to the Fifth Assessment Report of the Intergovernmental Panel on Climate Change*.

[36] Islam M. M., Sallu S., Hubacek K., Paavola J. (2014). Vulnerability of fishery-based livelihoods to the impacts of climate variability and change: insights from coastal Bangladesh. *Regional Environmental Change*.

[37] Moegni N., Rizki A., Prihantono G. (2014). Adaptation of marine capture Fishers in the face of climate change. *Jurnal Ekonomi dan Studi Pembangunan*.

[38] Cheung W. W. L., Pauly D., Laffoley D., dan Baxter J. M. (2016). Impacts and effects of ocean warming on marine fishes. *Explaining Ocean Warming: Causes, Scale, Effects and Consequences*.

[39] Patriana R., Satria A. (2015). Pola adaptasi nelayan terhadap perubahan iklim: studi kasus nelayan dusun ciawitali, desa pamotan, kecamatan kalipucang, kabupaten ciamis, jawa barat. *Jurnal Sosial Ekonomi Kelautan dan Perikanan*.

[40] Hernandez-Delgado E. A. (2015). The emerging threats of climate change on tropical coastal ecosystem services public health, local economies and livelihood sustainability of small islands: cumulative impacts and synergies. *Marine Pollution Bulletin*.

[41] Wibowo A., Satria A. (2015). Adaptation strategy of fishermen in small islands to the impacts of climate change (case: pulau panjang village, subi district, natuna regency, riau islands). *Sodality: Jurnal Sosiologi Pedesaan*.

[42] Nugroho B. D. A. (2016). *Global Climate Phenomena, Climate Change, and Their Impacts in Indonesia”*.

[43] Saut A. H. S., Argo T. A., Asirin, Adhitama P., Dodon (2016). Fishermen’s adaptation strategy to the impact of environmental change (Case study: utilization of marine fishing technology). *Jurnal Penataan Ruang*.

[44] Waldman K. B., Richardson R. B. (2018). Confronting tradeoffs between agricultural ecosystem services and adaptation to climate change in Mali. *Ecological Economics*.

[45] Marie M., Yirga F., Haile M., Tquabo F. (2020). Farmers’ choices and factors affecting adoption of climate change adaptation strategies: evidence from northwestern Ethiopia. *Heliyon*.

[46] Ahmed Z., Guha G. S., Shew A. M., Alam G. M. M. (2021). Climate change risk perceptions and agricultural adaptation strategies in vulnerable riverine char islands of Bangladesh. *Land Use Policy*.

[47] Sertse S. F., Khan N. A., Shah A. A., Liu Y., Naqvi S. A. A. (2021). Farm households’ perceptions and adaptation strategies to climate change risks and their determinants: evidence from Raya Azebo district, Ethiopia. *International Journal of Disaster Risk Reduction*.

[48] Adzawla W., Kudadze S., Mohammed A. R., Ibrahim I. I. (2019). Climate perceptions, farmers’ willingness-to-insure farms and resilience to climate change in Northern region, Ghana. *Environmental Development*.

[49] Hasan M. K., Kumar L. (2019). Comparison between meteorological data and farmer perceptions of climate change and vulnerability in relation to adaptation. *Journal of Environmental Management*.

[50] Singh S. (2020). Farmers’ perception of climate change and adaptation decisions: a micro-level evidence from Bundelkhand Region, India. *Ecological Indicators*.

[51] Aidoo D. C., Boateng S. D., Freeman C. K., Anaglo J. N. (2021). The effect of smallholder maize farmers’ perceptions of climate change on their adaptation strategies. *The Case Of Two Agro-Ecological Zones In Ghana” Heliyon*.

[52] Guodaar L., Bardsley D. K., Suh J. (2021). Integrating local perceptions with scientific evidence to understand climate change variability in northern Ghana: a mixed-methods approach. *Applied Geography*.

[53] Keshavarz M., Moqadas R. S. (2021). Assessing rural households’ resilience and adaptation strategies to climate variability and change. *Journal of Arid Environments*.

[54] Mairura F. S., Musafiri C. M., Kiboi M. N. (2021). Determinants of farmers’ perceptions of climate variability, mitigation, and adaptation strategies in the central highlands of Kenya. *Weather and Climate Extremes*.

[55] Suresh K., Khanal U., Wilson C., Managi S., Quayle A., Santhirakumar S. (2021). An economic analysis of agricultural adaptation to climate change impacts in Sri Lanka: an endogenous switching regression analysis. *Land Use Policy*.

[56] Talanow K., Topp E. N., Loos J., Martin-Lopez B. (2021). Farmers’ perceptions of climate change and adaptation strategies in South Africa’s Western Cape. *Journal of Rural Studies*.

[57] Midi H., Sarkar S. K., Rana S. (2010). Collinearity diagnostics of binary logistic regression model. *Journal of Interdisciplinary Mathematics*.

[58] Aldrian E. (2006). Long term rainfall trend of the Brantas catchment area, East Java. Indonesia. *The Indonesian Journal of Geography*.

[59] Saji N. H., Goswami B. N., Vinayachandran P. N., Yamagata T. (1999). A dipole mode in the tropical Indian ocean. *Nature*.

[60] Bmkg (2018). *Climate Online Data in Bengkulu Province, Meteorological, Climatological, and Geophysical Agency*.

[61] Maifizar A. (2018). Adaptation strategies based on Fishers’ local wisdom to changes in Aceh’s coastal ecosystem. *Community*.

[62] Mustaqim (2018). Adaptation of fishermen communities to changes in the ecosystem of the coastal area of Sabang Island. *Jurnal Ilmu Sosial dan Humaniora*.

[63] Macusi E. D., Macusi E. S., Jimenez L. A., Catam-isan J. P. (2020). Climate change vulnerability and perceived impacts on small-scale fisheries in eastern Mindanao. *Ocean and Coastal Management*.

[64] Gianelli I., Ortega L., Pittman J., Vasconcellos M., Defeo O. (2021). Harnessing scientific and local knowledge to face climate change in small-scale fisheries. *Global Environmental Change*.

[65] Begum M., Masud M. M., Alam L., Mokhtar M. B., Amir A. A. (2022). The adaptation behaviour of marine fishermen towards climate change and food security: an application of the theory of planned behaviour and health belief model. *Sustainability*.

[66] Clayton S., Devine-Wright P., Stern P. C. (2015). Psychological research and global climate change. *Nature Climate Change*.

[67] Bakun A., Black B. A., Bograd S. J. (2015). Anticipated effects of climate change on coastal upwelling ecosystems. *Current Climate Change Reports*.

[68] Odum E. P. (2004). *Ecology Basics, Translated by Tjahjono Samingan*.

[69] Ashoka Deepananda K. H. M., Macusi E. D. (2012). The changing climate and its implications to capture fisheries. *Journal of Nature Studies*.

[70] Muallil R. N., Geronimo R. C., Cleland D. (2011). Willingness to exit the artisanal fishery as a response to scenarios of declining catch or increasing monetary incentives. *Fisheries Research*.

[71] Macusi E. D., Katikiro R. E., Babaran R. P. (2017). The influence of economic factors in the change of fishing strategies of anchored FAD Fishers in the face of declining catch, General Santos City, Philippines. *Marine Policy*.

[72] Mulyasari G., Irham, Waluyati L. R., Suryantini A. (2018). Perceptions and local adaptation strategies to climate change of marine capture fishermen in Bengkulu Province, Indonesia. *IOP Conference Series: Earth and Environmental Science*.

[73] Wiyono E. S. (2013). Constraints and strategies for fishing operations for Bubu fishing gear in Muara Angke Jakarta. *Jurnal Ilmu Perikanan Tropis*.

[74] Zhang J., Fleming J., Goericke R. (2012). Fishermen’s perspectives on climate variability. *Marine Policy*.

[75] Nurlaili (2012). Adaptation strategy of bajo fishermen in facing climate change: study of bajo fishermen in sikka district, flores, east nusa tenggara. *Jurnal Masyarakat dan Budaya*.

[76] Subair S., Kolopaking L. M., Adiwibowo S., Pranowo M. B. (2015). Resiliensi komunitas dalam merespon perubahan iklim melalui strategi nafkah (skdnam. *Jurnal Sosial Ekonomi Kelautan dan Perikanan*.

