# Supplementary material for: Assessing Perceptions and Adaptation Responses to Climate Change among Small-Scale Fishery on the Northern Coastal of Bengkulu, Indonesia
Source: ScientificWorldJournal. 2023 Jan 17;2023:8770267. doi: 10.1155/2023/8770267 (PMC9873426; doi:10.1155/2023/8770267)
Supplement: Supplementary Materials — This article has figures to clarify information consisting of (1) a conceptual framework which describes the theoretical framework regarding the stages of implementing climate change adaptation; (2) a map of research locations explaining where this research was carried out specifically and visually; (3) taking and determining respondents using the multistage sampling technique; (4) socioeconomic characteristics of respondents including age, education, experience, household size, fishing income, and side job; and (5) trend indicators of climate change including annual rainfall, temperature, and humidity from 1985 to 2020. [file 8770267.f1.zip › Supplementary file 1.pdf]

Research areas	No.	Name of respondents	1	2
BENGKULU CITY	1	Detri Waldi		
	2	Sunardi		
	3	Darusalam	√	√
	4	Pes	√	√
	5	Sukri	√	√
	6	Candra		
	7	Agung	√	√
	8	Supriansuri	√	√
	9	Asmadi	√	√
	10	Hardi Sugito	√	√
	11	Sugianto	√	√
	12	Sandro	√	√
	13	Suardi	√	√
	14	Jon Hendra	√	√
	15	Lambro	√	√
	16	Madong	√	√
	17	Baharudin	√	√
	18	Ahmad Aziz	√	√
	19	Peprianto	√	√
	20	Fredy Yoki Antoni	√	√
	21	Joni Efendi	√	√
	22	Khairul	√	√
	23	Munadi	√	
	24	Edi		√
	25	Yansril		√
	26	Rizal	√	√
	27	Ujang	√	
	28	Judiko	√	√
	29	Ali Asman Tomat		√
	30	Demi	√	
	31	Dodi Agustin	√	√
	32	Sapran	√	√
	33	Rahmat	√	√
	34	Nasran Simamora	√	√
	35	Sumardianto	√	√
	36	Gusti Kurnama	√	√
	37	Kali Kunjoro	√	√
	38	Dahidres	√	√
	39	Umar	√	√
	40	Hakim	√	√
	41	Samsuri	√	√
	42	Ramlan	√	√
	43	Zulkarnain	√	√
	44	Herdi	√	√
	45	Ade Putra	√	√

	46	Ermin Suprapto	√	√
	47	Yas Budaya	√	√
	48	Israini	√	√
	49	Rahmat Nazri	√	√
	50	Irhandi	√	√
	51	Sadam Hussen	√	√
	52	Pebrianto Tanjung	√	√
	53	Sopian Sori	√	√
	54	Sumardi	√	√
	55	Liki Wiriansyah	√	√
	56	Roy Muhammad	√	√
	57	Jepriki	√	√
	58	Pajri Sinaga	√	√
	59	Agung Suryadi Putra	√	√
	60	Pelki Apriansyah Hutagalung	√	√
**NORTH BENGKULU [illegible]**	61	Heru		
	62	Rusdianto		
	63	Eri		√
	64	Hamdani		
	65	Repri		√
	66	Zahroni		√
	67	Julianto		√
	68	Oki		
	69	Gunawan		
	70	Joni		√
	71	Buyung		
	72	Ahmad Badri		
	73	Yendi		
	74	Misdi		
	75	Roslika		√
	76	Adi		
	77	Efendi		
	78	Hasal		
	79	Ahmad		
	80	Husen		
	81	Burhan		
	82	Soni		
	83	Basarudin		
	84	Angga		
	85	Eko		
	86	Hakim		
	87	Heri		
	88	Roni		
	89	Dwi		
	90	Darwis		
	91	aris	√	√
	92	darmawansyah	√	√

DISTRICT

93	Rudi irawan	√	√
94	muja	√	√
95	jusnan masa	√	√
96	Sahrudin	√	√
97	jopayano	√	√
98	Jumardi	√	√
99	arjoni	√	√
100	oki	√	√
101	hamdani	√	√
102	arvin anggara	√	√
103	Roskan	√	√
104	ilyas	√	√
105	Pendi	√	√
106	Alimunir	√	√
107	Alen	√	√
108	Saugani	√	√
109	Ipen	√	√
110	arjunes	√	√
111	Hanapi	√	√
112	Mujrimun	√	√
113	Sriyando	√	√
114	anwar	√	√
115	azwawi	√	√
116	Vebri	√	√
117	adi suwanto	√	√
118	Diran	√	√
119	Dedi	√	√
120	fajri	√	√
121	Elpis		√
122	Azhar		√
123	Edi Aryanto		√
124	Edi Geleng		√
125	Mulyanto		√
126	Dedi		√
127	Pausi		√
128	Mulyadi		√
129	Rahmat		√
130	Indra Saputra		√
131	Agusri		√
132	Tulang		√
133	Buyung G		√
134	Agus		√
135	Kurik		√
136	Yan		√
137	Ridho		√
138	Yoga Pratama		√
139	Hendra		√

MUKOMUKO DISTRICT

140	Sapriyanto		√
141	Abdul Mulis		√
142	Yogi		√
143	Wanda		√
144	Suman		√
145	Rio		√
146	Agus R		√
147	Bambang		√
148	Hendra Saputra		√
149	Nopri		√
150	Joni		√
151	Syafi'i		√
152	Sutrisno		√
153	M Fauzi		√
154	Iwaldi		√
155	Sudirman		√
156	Vio		√
157	Hendri		√
158	Antoni		√
159	Sapar		√
160	Rahmat Syukur		√
161	Idep		√
162	Rendi		√
163	Dedi		√
164	Eddy N		√
165	Rendy Aprianto		√
166	Iwal		√
167	Danil		√
168	Thamrani		√
169	Adek		√
170	Ucok		√
171	Ono		√
172	Parel		√
173	Aan		√
174	Kul		√
175	Cucun		√
176	Lodi		√
177	Sanusi		√
178	Ujang		√
179	Ap		√
180	Bono		√
181	Edi Suito		√
182	Sami Hermawan		√
183	Suadi Efendi		√
184	Hendra		√
185	Muskardi		√
186	Antoni		√

SOUTH BENGKULU DISTRICT

187	Junaidi		√
188	Edi		√
189	Utung		√
190	Julian Efendi		√
191	Yahudi		√
192	Dona		√
193	Mawan		√
194	Julian Agusman		√
195	Dawar		√
196	M. Musni		√
197	Endi		√
198	Joyo		√
199	Yayan		√
200	Tomi		√
201	Merza		√
202	Wiwin		√
203	Bambang		√
204	Atang		√
205	Eka		√
206	Febry		√
207	Mairon		√
208	Dedi		√
209	Junaidi Eko		√
210	Sirat		√
211	Suhaybi		√
212	Hopla		√
213	Otong Lenong		√
214	Jon		√
215	Aliman Efendi		√
216	Basuki		√
217	Sukman		√
218	Suharyanto		√
219	Supiasari		√
220	Dayat Efendi		√
221	Romi		√
222	Ipul		√
223	Trio		√
224	Alam Sunarjo		√
225	Tomi		√
226	Peri		√
227	Pebi		√
228	Herman		√
229	Sasan		√
230	Rudi		√
231	Pendi		√
232	Pino		√
233	Wiwin		√

	234	Riskan		√
	235	Latif		√
	236	Cecep		√
	237	Maman		√
	238	Yayan		√
	239	Indra		√
	240	Iwan		√
**KAUR DISTRICT**	241	Afrizal		√
	242	Merwan		√
	243	Sirat Sudarman		√
	244	Farizal Latif		√
	245	Nosep		√
	246	Syukur		
	247	Tarbani		
	248	Meliansyah		√
	249	Heri Bahtiar		
	250	Muhammad Effendi		
	251	Agus		√
	252	Andre		√
	253	Suparyadi		√
	254	Ali Imron		
	255	Mahdiantomi		√
	256	Japri		√
	257	Jamalludin		
	258	Herisusanti		√
	259	Basarudin		√
	260	Supriyadi		√
	261	Samsul Bahri		√
	262	Andika		
	263	Muhammad ali		√
	264	Nasrudin		√
	265	Aan Saputra		
	266	Sulaiman		
	267	Hidayat		√
	268	Zainal Arifin		√
	269	Badrun		√
	270	Ali Usman		√
	271	Munizar		√
	272	Zinul		√
	273	Firman		√
	274	Kauri		√
	275	Iwan		√
	276	Mursi		√
	277	Hasan		√
	278	Bahsan		√
	279	Hendra		√
	280	Hasan N		√

	281	Apen		√
	282	Sumardi		√
	283	Alman		√
	284	Elman		√
	285	Sopian		√
	286	Yudi		√
	287	Aris		√
	288	Jonli		√
	289	Safii		√
	290	Arsan		√
	291	Muherwan		√
	292	Pendri		√
	293	Bait		√
	294	Salman		√
	295	Yudi		√
	296	Basarudin		√
	297	Sapwan		√
	298	Ruslan		√
	299	Joni		√
	300	Merdi		√

Adaptation Strategies									
3	4	5	6	7	8	9	10	11	12
		√					√	√	√
√		√			√		√	√	√
								√	√
	√		√					√	√
							√		
√		√			√		√		√
√		√					√	√	√
							√	√	√
√		√			√		√		√
		√							√
√	√	√					√	√	√
√	√	√	√	√	√		√	√	√
√	√	√						√	√
√	√	√	√	√			√	√	√
√	√	√	√	√			√	√	√
√	√		√	√			√	√	√
		√						√	√
									√
									√
							√		
									√
		√							
	√	√					√	√	√
									√
√	√	√	√	√	√	√	√	√	√
		√	√	√			√	√	√
		√					√		
√	√	√	√	√	√	√		√	√
√	√	√	√	√	√	√		√	√
√	√	√	√	√	√	√		√	√
√	√	√	√	√	√	√		√	√
√	√	√	√	√	√	√		√	√
√	√	√	√	√	√	√		√	√
√	√	√	√	√	√	√		√	√
√	√	√	√	√	√	√		√	√
√	√	√	√	√	√	√		√	√
√	√	√	√	√	√	√		√	√
√	√	√	√	√	√	√		√	√
√	√	√	√	√	√	√		√	√
√	√	√	√	√	√	√		√	√
√	√	√	√	√	√	√		√	√
√	√	√	√	√	√	√		√	√
√	√	√	√	√	√	√		√	√

√	√	√	√	√	√	√		√	√
√	√	√	√	√	√	√		√	√
√	√	√	√	√	√	√		√	√
√	√	√	√	√	√	√		√	√
√	√	√	√	√	√	√		√	√
√	√	√	√	√	√	√		√	√
√	√	√	√	√	√	√		√	√
√	√	√	√	√	√	√		√	√
√	√	√	√	√	√	√		√	√
√	√	√	√	√	√	√		√	√
√	√	√	√	√	√	√		√	√
√	√	√	√	√	√	√		√	√
√	√	√	√	√	√	√		√	√
√	√	√	√	√	√	√		√	√
√	√	√	√	√	√	√		√	√
									√
									√
			√					√	√
								√	√
			√					√	√
								√	√
					√	√	√		
									√
								√	
									√
						√			
						√			
						√	√		
						√			
						√			
						√			
						√			
						√			
						√			
√			√	√		√		√	√
√			√	√				√	√

√			√	√		√		√	√
√			√	√		√		√	√
√			√	√				√	√
√			√	√		√		√	√
√			√	√		√		√	√
√			√	√				√	√
√			√	√		√		√	√
√			√	√		√		√	√
√			√	√		√		√	√
√			√	√				√	√
√			√	√		√		√	√
√			√	√				√	√
√			√	√				√	√
√			√	√				√	√
√			√	√				√	√
√			√	√				√	√
√			√	√				√	√
√			√	√				√	√
√			√	√				√	√
√			√	√		√		√	√
√			√	√				√	√
√			√	√				√	√
√			√	√				√	√
√			√	√				√	√
√			√	√				√	√
√			√	√				√	√
√			√	√		√		√	√
√			√	√				√	√
	√	√	√	√					√
	√	√	√	√					√
	√	√	√	√					√
	√	√	√	√					√
	√	√	√	√					√
	√	√	√	√					√
	√	√	√	√					√
	√	√	√	√					√
	√	√	√	√					√
	√	√	√	√					√
	√	√	√	√					√
	√	√	√	√					√
	√	√	√	√					√
	√	√	√	√					√
	√	√	√	√					√
	√	√	√	√					√
	√	√	√	√					√
	√	√	√	√					√
	√	√	√	√					√

	√	√	√	√					√
	√	√	√	√					√
	√	√	√	√					√
	√	√	√	√					√
	√	√	√	√					√
	√	√	√	√					√
	√	√	√	√					√
	√	√	√	√					√
	√	√	√	√					√
	√	√	√	√					√
	√	√	√	√					√
	√	√	√	√					√
	√	√	√	√					√
	√	√	√	√					√
	√	√	√	√					√
	√	√	√	√					√
	√	√	√	√					√
	√	√	√	√					√
	√	√	√	√					√
	√	√	√	√					√
	√	√	√	√					√
	√	√	√	√					√
	√	√	√	√					√
	√	√	√	√					√
	√	√	√	√					√
	√	√	√	√					√
	√	√	√	√					√
	√	√	√	√					√
	√	√	√	√					√
	√	√	√	√					√
	√	√	√	√					√
	√	√	√	√					√
	√	√	√	√					√
	√	√	√	√					√
	√	√	√	√					√
	√	√	√	√					√
	√	√	√	√					√
	√	√	√	√					√
	√	√	√	√					√
	√	√	√	√					√
	√	√	√	√					√
		√	√	√			√	√	√
		√	√	√			√	√	√
		√	√	√			√	√	√
		√	√	√			√	√	√
		√	√	√			√	√	√
		√	√	√			√	√	√

		√	√	√			√	√	√
		√	√	√			√	√	√
		√	√	√			√	√	√
		√	√	√			√	√	√
		√	√	√			√	√	√
		√	√	√			√	√	√
		√	√	√			√	√	√
		√	√	√			√	√	√
		√	√	√			√	√	√
		√	√	√			√	√	√
		√	√	√			√	√	√
		√	√	√			√	√	√
		√	√	√			√	√	√
		√	√	√			√	√	√
		√	√	√			√	√	√
		√	√	√			√	√	√
		√	√	√			√	√	√
		√	√	√			√	√	√
		√	√	√			√	√	√
		√	√	√			√	√	√
		√	√	√			√	√	√
		√	√	√			√	√	√
		√	√	√			√	√	√
		√	√	√			√	√	√
	√	√	√			√	√	√	√
	√	√	√			√	√	√	√
	√	√	√			√	√	√	√
	√	√	√			√	√	√	√
	√	√	√			√	√	√	√
	√	√	√			√	√	√	√
	√	√	√			√	√	√	√
	√	√	√			√	√	√	√
	√	√	√			√	√	√	√
	√	√	√			√	√	√	√
	√	√	√			√	√	√	√
	√	√	√			√	√	√	√
	√	√	√			√	√	√	√
	√	√	√			√	√	√	√
	√	√	√			√	√	√	√
	√	√	√			√	√	√	√
	√	√	√			√	√	√	√
	√	√	√			√	√	√	√
	√	√	√			√	√	√	√
	√	√	√			√	√	√	√
	√	√	√			√	√	√	√
	√	√	√			√	√	√	√
	√	√	√			√	√	√	√

	√	√	√			√	√	√	√
	√	√	√			√	√	√	√
	√	√	√			√	√	√	√
	√	√	√			√	√	√	√
	√	√	√			√	√	√	√
	√	√	√			√	√	√	√
	√	√	√			√	√	√	√
√		√	√	√				√	√
√			√	√				√	√
√			√	√				√	√
√			√	√				√	√
			√	√				√	√
								√	√
			√	√				√	√
	√		√	√				√	√
			√	√				√	√
			√	√				√	√
√	√		√	√			√	√	√
			√	√			√	√	√
			√	√				√	√
			√	√				√	√
								√	√
√			√	√				√	√
								√	√
			√	√			√	√	√
			√	√				√	√
			√	√			√	√	√
			√	√				√	√
								√	√
			√	√				√	√
			√	√				√	√
							√	√	√
			√	√			√	√	√
			√	√			√	√	√
			√	√			√	√	√
			√	√			√	√	√
			√	√			√	√	√
√			√					√	√
√			√					√	√
√			√					√	√
√			√					√	√
√			√					√	√
√			√					√	√
√			√					√	√
√			√					√	√
√			√					√	√
√			√					√	√

√			√					√	√
√			√					√	√
√			√					√	√
√			√					√	√
√			√					√	√
√			√					√	√
√			√					√	√
√			√					√	√
√			√					√	√
√			√					√	√
√			√					√	√
√			√					√	√
√			√					√	√
√			√					√	√
√			√					√	√
√			√					√	√
√			√					√	√
√			√					√	√
√			√					√	√
√			√					√	√

13	14	15	16	Number of adaptation	Level of adaptation	Adaptation responses
√	√	√		7	0.44	0
				0	0.00	0
√	√			10	0.63	1
√				5	0.31	0
√	√			8	0.50	0
				0	0.00	0
√				4	0.25	0
√	√			9	0.56	1
√				8	0.50	0
√	√	√		8	0.50	0
√	√			9	0.56	1
√				5	0.31	0
				2	0.13	0
√	√			10	0.63	1
√				12	0.75	1
√	√	√		10	0.63	1
√	√	√	√	14	0.88	1
√	√			12	0.75	1
√				10	0.63	1
√				6	0.38	0
√				4	0.25	0
√				4	0.25	0
√				3	0.19	0
√				3	0.19	0
√				3	0.19	0
√	√			9	0.56	1
√				3	0.19	0
√	√			14	0.88	1
√				8	0.50	0
√	√			5	0.31	0
√	√			13	0.81	1
√	√			13	0.81	1
√	√			13	0.81	1
√	√			13	0.81	1
√	√	√		14	0.88	1
√	√			13	0.81	1
√	√	√		14	0.88	1
√	√	√	√	15	0.94	1
√	√			13	0.81	1
√	√	√		14	0.88	1
√	√	√		14	0.88	1
√	√	√		14	0.88	1
√	√	√		14	0.88	1
√	√	√		14	0.88	1
√	√	√		14	0.88	1

√	√	√		14	0.88	1
√	√	√		14	0.88	1
√	√	√		14	0.88	1
√	√	√		14	0.88	1
√	√	√		14	0.88	1
√	√	√		14	0.88	1
√	√	√		14	0.88	1
√	√	√		14	0.88	1
√	√	√		14	0.88	1
√	√	√		14	0.88	1
√	√	√		14	0.88	1
√	√	√		14	0.88	1
√	√	√		14	0.88	1
√	√	√		14	0.88	1
√	√	√		14	0.88	1
		√		2	0.13	0
	√	√		3	0.19	0
	√	√		6	0.38	0
				2	0.13	0
	√			4	0.25	0
	√	√		5	0.31	0
	√	√		6	0.38	0
	√			2	0.13	0
				1	0.06	0
				1	0.06	0
		√		1	0.06	0
				0	0.00	0
		√		1	0.06	0
			√	1	0.06	0
√			√	4	0.25	0
	√			2	0.13	0
	√			1	0.06	0
				1	0.06	0
				2	0.13	0
				1	0.06	0
				1	0.06	0
				1	0.06	0
	√			2	0.13	0
				1	0.06	0
	√			2	0.13	0
				0	0.00	0
				0	0.00	0
				0	0.00	0
				0	0.00	0
				0	0.00	0
	√			9	0.56	1
	√			8	0.50	0

	√		√	10	0.63	1
	√			9	0.56	1
	√	√		9	0.56	1
	√			9	0.56	1
	√			9	0.56	1
	√			8	0.50	0
	√			9	0.56	1
	√			9	0.56	1
	√			9	0.56	1
	√			8	0.50	0
	√	√	√	11	0.69	1
	√			8	0.50	0
	√	√		9	0.56	1
	√	√	√	10	0.63	1
	√			8	0.50	0
	√	√		9	0.56	1
	√			8	0.50	0
	√			8	0.50	0
	√			8	0.50	0
	√			9	0.56	1
	√		√	9	0.56	1
	√		√	9	0.56	1
	√		√	9	0.56	1
	√			8	0.50	0
	√			8	0.50	0
	√			8	0.50	0
	√			9	0.56	1
	√		√	9	0.56	1
√	√		√	9	0.56	1
√	√		√	9	0.56	1
√	√		√	9	0.56	1
√	√		√	9	0.56	1
√	√		√	9	0.56	1
√	√		√	9	0.56	1
√	√		√	9	0.56	1
√	√		√	9	0.56	1
√	√		√	9	0.56	1
√	√		√	9	0.56	1
√	√		√	9	0.56	1
√	√		√	9	0.56	1
√	√		√	9	0.56	1
√	√		√	9	0.56	1
√	√		√	9	0.56	1
√	√		√	9	0.56	1
√	√		√	9	0.56	1
√	√		√	9	0.56	1
√	√		√	9	0.56	1

√	√		√	9	0.56	1
√	√		√	9	0.56	1
√	√		√	9	0.56	1
√	√		√	9	0.56	1
√	√		√	9	0.56	1
√	√		√	9	0.56	1
√	√		√	9	0.56	1
√	√		√	9	0.56	1
√	√		√	9	0.56	1
√	√		√	9	0.56	1
√	√		√	9	0.56	1
√	√		√	9	0.56	1
√	√		√	9	0.56	1
√	√		√	9	0.56	1
√	√		√	9	0.56	1
√	√		√	9	0.56	1
√	√		√	9	0.56	1
√	√		√	9	0.56	1
√	√		√	9	0.56	1
√	√		√	9	0.56	1
√	√		√	9	0.56	1
√	√		√	9	0.56	1
√	√		√	9	0.56	1
√	√		√	9	0.56	1
√	√		√	9	0.56	1
√	√		√	9	0.56	1
√	√		√	9	0.56	1
√	√		√	9	0.56	1
√	√		√	9	0.56	1
√	√		√	9	0.56	1
√	√		√	9	0.56	1
√	√		√	9	0.56	1
√	√		√	9	0.56	1
√	√		√	9	0.56	1
√	√		√	9	0.56	1
√	√		√	9	0.56	1
√	√		√	9	0.56	1
√	√		√	9	0.56	1
√	√		√	9	0.56	1
√	√		√	9	0.56	1
√	√		√	9	0.56	1
√	√	√	√	11	0.69	1
√	√	√	√	11	0.69	1
√	√	√	√	11	0.69	1
√	√	√	√	11	0.69	1
√	√	√	√	11	0.69	1
√	√	√	√	11	0.69	1

√	√	√	√	11	0.69	1
√	√	√	√	11	0.69	1
√	√	√	√	11	0.69	1
√	√	√	√	11	0.69	1
√	√	√	√	11	0.69	1
√	√	√	√	11	0.69	1
√	√	√	√	11	0.69	1
√	√	√	√	11	0.69	1
√	√	√	√	11	0.69	1
√	√	√	√	11	0.69	1
√	√	√	√	11	0.69	1
√	√	√	√	11	0.69	1
√	√	√	√	11	0.69	1
√	√	√	√	11	0.69	1
√	√	√	√	11	0.69	1
√	√	√	√	11	0.69	1
√	√	√	√	11	0.69	1
√	√	√	√	11	0.69	1
√	√	√	√	11	0.69	1
√	√	√	√	11	0.69	1
√	√	√	√	11	0.69	1
√	√	√	√	11	0.69	1
√	√	√	√	11	0.69	1
√	√	√	√	11	0.69	1
√	√	√	√	12	0.75	1
√	√	√	√	12	0.75	1
√	√	√	√	12	0.75	1
√	√	√	√	12	0.75	1
√	√	√	√	12	0.75	1
√	√	√	√	12	0.75	1
√	√	√	√	12	0.75	1
√	√	√	√	12	0.75	1
√	√	√	√	12	0.75	1
√	√	√	√	12	0.75	1
√	√	√	√	12	0.75	1
√	√	√	√	12	0.75	1
√	√	√	√	12	0.75	1
√	√	√	√	12	0.75	1
√	√	√	√	12	0.75	1
√	√	√	√	12	0.75	1
√	√	√	√	12	0.75	1
√	√	√	√	12	0.75	1
√	√	√	√	12	0.75	1
√	√	√	√	12	0.75	1
√	√	√	√	12	0.75	1
√	√	√	√	12	0.75	1
√	√	√	√	12	0.75	1

√	√	√	√	12	0.75	1
√	√	√	√	12	0.75	1
√	√	√	√	12	0.75	1
√	√	√	√	12	0.75	1
√	√	√	√	12	0.75	1
√	√	√	√	12	0.75	1
√	√	√	√	12	0.75	1
√	√		√	10	0.63	1
√	√	√		9	0.56	1
√	√	√		9	0.56	1
√	√	√		9	0.56	1
√	√	√		9	0.56	1
		√		3	0.19	0
	√	√		6	0.38	0
√	√	√		9	0.56	1
				4	0.25	0
				4	0.25	0
√	√	√		11	0.69	1
√	√	√		9	0.56	1
√	√	√		8	0.50	0
√	√			6	0.38	0
				6	0.38	0
√	√	√		9	0.56	1
				2	0.13	0
		√		6	0.38	0
√	√	√		8	0.50	0
√	√	√		9	0.56	1
√	√	√		8	0.50	0
		√		3	0.19	0
√	√			7	0.44	0
√	√	√	√	9	0.56	1
		√		4	0.25	0
			√	6	0.38	0
		√		7	0.44	0
√	√	√		9	0.56	1
√	√	√	√	10	0.63	1
√	√			8	0.50	0
√	√			7	0.44	0
√	√			7	0.44	0
√	√			7	0.44	0
√	√			7	0.44	0
√	√			7	0.44	0
√	√			7	0.44	0
√	√			7	0.44	0
√	√			7	0.44	0
√	√			7	0.44	0
√	√			7	0.44	0

√	√			7	0.44	0
				5	0.31	0
√	√			7	0.44	0
√	√			7	0.44	0
√	√			7	0.44	0
√	√			7	0.44	0
√	√			7	0.44	0
√	√			7	0.44	0
√	√			7	0.44	0
√	√			7	0.44	0
√	√			7	0.44	0
√	√			7	0.44	0
√	√			7	0.44	0
				5	0.31	0
				5	0.31	0
√	√			7	0.44	0
√	√			7	0.44	0
√	√			7	0.44	0
√	√			7	0.44	0
				5	0.31	0
