# Supplementary material for: Assessing Perceptions and Adaptation Responses to Climate Change among Small-Scale Fishery on the Northern Coastal of Bengkulu, Indonesia
Source: ScientificWorldJournal. 2023 Jan 17;2023:8770267. doi: 10.1155/2023/8770267 (PMC9873426; doi:10.1155/2023/8770267)
Supplement: Supplementary Materials — This article has figures to clarify information consisting of (1) a conceptual framework which describes the theoretical framework regarding the stages of implementing climate change adaptation; (2) a map of research locations explaining where this research was carried out specifically and visually; (3) taking and determining respondents using the multistage sampling technique; (4) socioeconomic characteristics of respondents including age, education, experience, household size, fishing income, and side job; and (5) trend indicators of climate change including annual rainfall, temperature, and humidity from 1985 to 2020. [file 8770267.f1.zip › Supplementary file 2.pdf]

Research areas	No.	Name of respondents	Adaptation Responses	Age
BENGKULU CITY	1	Detri Waldi	0	50
	2	Sunardi	0	54
	3	Darusalam	1	43
	4	Pes	0	47
	5	Sukri	0	43
	6	Candra	0	36
	7	Agung	0	42
	8	Supriansuri	1	50
	9	Asmadi	0	52
	10	Hardi Sugito	0	32
	11	Sugianto	1	32
	12	Sandro	0	42
	13	Suardi	0	57
	14	Jon Hendra	1	40
	15	Lambro	1	50
	16	Madong	1	40
	17	Baharudin	1	53
	18	Ahmad Aziz	1	59
	19	Peprianto	1	27
	20	Fredy Yoki Antoni	0	38
	21	Joni Efendi	0	34
	22	Khairul	0	59
	23	Munadi	0	51
	24	Edi	0	53
	25	Yansril	0	42
	26	Rizal	1	40
	27	Ujang	0	60
	28	Judiko	1	42
	29	Ali Asman Tomat	0	60
	30	Demi	0	37
	31	Dodi Agustin	1	36
	32	Sapran	1	41
	33	Rahmat	1	54
	34	Nasran Simamora	1	54
	35	Sumardianto	1	50
	36	Gusti Kurnama	1	60
	37	Kali Kunjoro	1	32
	38	Dahidres	1	50
	39	Umar	1	30
	40	Hakim	1	55
	41	Samsuri	1	54
	42	Ramlan	1	43
	43	Zulkarnain	1	49
	44	Herdi	1	40
	45	Ade Putra	1	27

	46	Ermin Suprapto	1	40
	47	Yas Budaya	1	51
	48	Israini	1	45
	49	Rahmat Nazri	1	47
	50	Irhandi	1	42
	51	Sadam Hussen	1	31
	52	Pebrianto Tanjung	1	37
	53	Sopian Sori	1	54
	54	Sumardi	1	56
	55	Liki Wiriansyah	1	34
	56	Roy Muhammad	1	36
	57	Jepriki	1	29
	58	Pajri Sinaga	1	63
	59	Agung Suryadi Putra	1	20
	60	Pelki Apriansyah Hutagalung	1	31
**NORTH BENGKULU [illegible]**	61	Heru	0	37
	62	Rusdianto	0	38
	63	Eri	0	30
	64	Hamdani	0	58
	65	Repri	0	20
	66	Zahroni	0	37
	67	Julianto	0	39
	68	Oki	0	20
	69	Gunawan	0	38
	70	Joni	0	23
	71	Buyung	0	39
	72	Ahmad Badri	0	49
	73	Yendi	0	43
	74	Misdi	0	40
	75	Roslika	0	56
	76	Adi	0	25
	77	Efendi	0	51
	78	Hasal	0	43
	79	Ahmad	0	20
	80	Husen	0	27
	81	Burhan	0	36
	82	Soni	0	46
	83	Basarudin	0	55
	84	Angga	0	26
	85	Eko	0	28
	86	Hakim	0	30
	87	Heri	0	22
	88	Roni	0	40
	89	Dwi	0	25
	90	Darwis	0	31
	91	aris	1	14
	92	darmawansyah	0	47

DISTRICT

93	Rudi irawan	1	40
94	muja	1	28
95	jusnan masa	1	50
96	Sahrudin	1	70
97	jopayano	1	18
98	Jumardi	0	29
99	arjoni	1	40
100	oki	1	27
101	hamdani	1	31
102	arvin anggara	0	29
103	Roskan	1	70
104	ilyas	0	52
105	Pendi	1	35
106	Alimunir	1	53
107	Alen	0	21
108	Saugani	1	56
109	Ipen	0	29
110	arjunes	0	43
111	Hanapi	0	56
112	Mujrimun	1	46
113	Sriyando	1	52
114	anwar	1	50
115	azwawi	1	71
116	Vebri	0	22
117	adi suwanto	0	29
118	Diran	0	75
119	Dedi	1	30
120	fajri	1	50
121	Elpis	1	38
122	Azhar	1	50
123	Edi Aryanto	1	28
124	Edi Geleng	1	75
125	Mulyanto	1	40
126	Dedi	1	31
127	Pausi	1	45
128	Mulyadi	1	50
129	Rahmat	1	54
130	Indra Saputra	1	45
131	Agusri	1	45
132	Tulang	1	35
133	Buyung G	1	38
134	Agus	1	54
135	Kurik	1	40
136	Yan	1	40
137	Ridho	1	28
138	Yoga Pratama	1	27
139	Hendra	1	42

MUKOMUKO DISTRICT

140	Sapriyanto	1	50
141	Abdul Mulis	1	70
142	Yogi	1	30
143	Wanda	1	31
144	Suman	1	56
145	Rio	1	32
146	Agus R	1	35
147	Bambang	1	45
148	Hendra Saputra	1	32
149	Nopri	1	29
150	Joni	1	40
151	Syafi'i	1	65
152	Sutrisno	1	33
153	M Fauzi	1	36
154	Iwaldi	1	37
155	Sudirman	1	47
156	Vio	1	35
157	Hendri	1	35
158	Antoni	1	41
159	Sapar	1	32
160	Rahmat Syukur	1	36
161	Idep	1	28
162	Rendi	1	30
163	Dedi	1	30
164	Eddy N	1	40
165	Rendy Aprianto	1	42
166	Iwal	1	38
167	Danil	1	30
168	Thamrani	1	55
169	Adek	1	40
170	Ucok	1	33
171	Ono	1	36
172	Parel	1	28
173	Aan	1	37
174	Kul	1	35
175	Cucun	1	35
176	Lodi	1	40
177	Sanusi	1	38
178	Ujang	1	42
179	Ap	1	33
180	Bono	1	30
181	Edi Suito	1	38
182	Sami Hermawan	1	40
183	Suadi Efendi	1	46
184	Hendra	1	30
185	Muskardi	1	62
186	Antoni	1	55

SOUTH BENGKULU DISTRICT

187	Junaidi	1	45
188	Edi	1	30
189	Utung	1	45
190	Julian Efendi	1	46
191	Yahudi	1	42
192	Dona	1	30
193	Mawan	1	35
194	Julian Agusman	1	46
195	Dawar	1	38
196	M. Musni	1	46
197	Endi	1	35
198	Joyo	1	35
199	Yayan	1	43
200	Tomi	1	46
201	Merza	1	46
202	Wiwin	1	46
203	Bambang	1	55
204	Atang	1	38
205	Eka	1	46
206	Febry	1	35
207	Mairon	1	30
208	Dedi	1	30
209	Junaidi Eko	1	38
210	Sirat	1	52
211	Suhaybi	1	35
212	Hopla	1	45
213	Otong Lenong	1	34
214	Jon	1	39
215	Aliman Efendi	1	50
216	Basuki	1	46
217	Sukman	1	37
218	Suharyanto	1	40
219	Supiasari	1	40
220	Dayat Efendi	1	39
221	Romi	1	38
222	Ipul	1	40
223	Trio	1	35
224	Alam Sunarjo	1	43
225	Tomi	1	37
226	Peri	1	35
227	Pebi	1	35
228	Herman	1	48
229	Sasan	1	40
230	Rudi	1	38
231	Pendi	1	35
232	Pino	1	37
233	Wiwin	1	40

	234	Riskan	1	37
	235	Latif	1	35
	236	Cecep	1	34
	237	Maman	1	36
	238	Yayan	1	48
	239	Indra	1	38
	240	Iwan	1	35
KAUR DISTRICT	241	Afrizal	1	45
	242	Merwan	1	41
	243	Sirat Sudarman	1	50
	244	Farizal Latif	1	40
	245	Nosep	1	34
	246	Syukur	0	60
	247	Tarbani	0	51
	248	Meliansyah	1	30
	249	Heri Bahtiar	0	31
	250	Muhammad Effendi	0	40
	251	Agus	1	35
	252	Andre	1	35
	253	Suparyadi	0	41
	254	Ali Imron	0	38
	255	Mahdiantomi	0	43
	256	Japri	1	38
	257	Jamalludin	0	70
	258	Herisusanti	0	51
	259	Basarudin	0	39
	260	Supriyadi	1	42
	261	Samsul Bahri	0	41
	262	Andika	0	29
	263	Muhammad ali	0	30
	264	Nasrudin	1	36
	265	Aan Saputra	0	43
	266	Sulaiman	0	40
	267	Hidayat	0	43
	268	Zainal Arifin	1	38
	269	Badrun	1	45
	270	Ali Usman	0	42
	271	Munizar	0	39
	272	Zinul	0	53
	273	Firman	0	25
	274	Kauri	0	53
	275	Iwan	0	32
	276	Mursi	0	40
	277	Hasan	0	39
	278	Bahsan	0	46
	279	Hendra	0	29
	280	Hasan N	0	40

	281	Apen	0	50
	282	Sumardi	0	57
	283	Alman	0	58
	284	Elman	0	50
	285	Sopian	0	45
	286	Yudi	0	36
	287	Aris	0	28
	288	Jonli	0	43
	289	Safii	0	50
	290	Arsan	0	52
	291	Muherwan	0	49
	292	Pendri	0	30
	293	Bait	0	41
	294	Salman	0	40
	295	Yudi	0	55
	296	Basarudin	0	52
	297	Sapwan	0	40
	298	Ruslan	0	51
	299	Joni	0	40
	300	Merdi	0	32

Years of schooling	Fishing experience	Household size	Fishing income	Boat power	Perceived climate change	Perceived climate impacts	Access to climate information
6	30	4	100.73	24	3.73	3.41	0
6	14	3	171.24	26	2.82	3.00	1
12	23	3	512.96	24	3.00	3.27	0
6	15	1	130.57	24	2.91	3.91	0
6	15	2	261.15	24	3.18	3.45	0
9	5	3	52.23	25	3.36	3.86	0
9	15	3	167.88	26	4.55	3.00	1
6	28	3	149.23	26	3.55	3.00	0
9	25	1	100.73	26	4.00	4.00	1
6	5	4	251.82	26	3.64	3.50	0
6	10	3	167.88	26	3.09	3.27	0
6	10	3	503.64	26	3.73	3.36	0
6	20	3	391.72	26	4.18	3.00	1
6	15	4	587.58	26	3.27	3.45	0
6	20	3	1,203.13	26	3.18	3.00	0
6	15	3	419.70	26	4.09	3.50	1
6	20	4	1,119.19	26	3.18	4.45	0
6	29	3	447.68	26	3.27	4.00	1
6	5	0	2,891.27	26	3.45	3.64	1
6	10	4	475.66	26	4.36	5.00	0
9	7	2	1,175.15	26	3.82	3.68	1
6	25	3	671.52	26	4.27	3.86	0
0	20	4	279.80	26	3.09	3.45	0
0	15	4	335.76	28	3.36	3.68	0
0	25	4	27.98	28	3.18	5.00	0
0	15	2	214.51	24	3.55	4.82	0
0	40	1	59.69	26	3.18	3.00	1
6	14	4	699.50	28	3.91	4.55	0
0	35	2	67.15	26	2.91	3.00	0
12	20	3	643.54	26	3.73	4.36	0
9	15	3	48.68	26	3.18	3.64	0
6	24	3	31.34	26	3.73	4.00	0
9	40	4	54.09	26	4.09	4.00	0
9	30	3	49.66	26	3.73	3.00	0
6	38	4	51.30	26	3.73	4.00	1
9	40	6	51.30	26	3.82	3.45	1
6	12	3	55.96	26	3.82	3.55	1
9	20	2	69.95	26	4.09	3.55	0
6	8	3	93.27	26	3.91	3.55	1
6	30	4	48.50	26	3.45	3.45	1
6	30	5	29.38	26	3.45	3.50	0
6	20	2	46.63	26	3.09	3.50	1
9	23	8	35.25	26	3.73	3.50	0
6	20	2	34.97	26	3.73	3.50	0
12	9	1	36.51	26	3.45	3.50	0

9	18	7	41.04	26	3.36	3.45	0
6	10	3	26.58	26	3.73	3.45	0
6	20	3	36.72	26	3.45	3.50	0
6	20	4	33.58	26	3.45	3.50	0
6	20	4	27.98	26	3.27	3.59	1
9	10	3	31.90	26	3.73	3.55	0
6	15	0	20.52	26	3.64	3.45	1
6	30	6	33.58	26	3.45	3.45	1
6	30	2	41.97	26	3.45	3.50	1
6	13	2	39.17	26	4.09	3.55	1
6	15	3	30.78	26	3.45	3.55	0
9	10	2	33.58	26	3.36	3.55	0
6	40	3	55.96	26	3.73	3.45	1
12	1	2	33.58	26	3.73	3.45	1
6	14	2	25.65	26	3.36	3.50	1
9	10	3	1,479.82	15	4.55	4.50	0
9	15	3	1,134.12	15	4.55	4.14	1
9	2	2	1,479.82	15	4.55	4.09	1
0	21	1	1,701.18	15	4.64	4.32	0
12	2	0	2,835.30	15	4.18	4.55	1
12	10	4	2,835.30	25	4.27	4.45	1
9	20	4	1,479.82	15	4.64	4.68	0
12	2	0	1,417.65	15	4.27	4.59	1
12	25	4	1,454.95	15	4.36	4.68	0
6	2	2	2,126.47	15	4.55	4.50	1
6	24	3	1,417.65	15	4.64	3.77	0
6	30	2	2,367.71	15	4.00	4.00	1
12	28	4	1,417.65	15	4.64	4.77	1
6	2	4	1,417.65	15	4.64	3.95	0
6	40	0	1,134.12	15	4.45	4.55	0
6	10	2	1,417.65	15	4.73	4.82	0
6	33	2	2,126.47	15	4.82	4.14	1
12	25	3	739.91	15	4.73	4.73	1
6	7	0	2,835.30	15	4.82	4.14	1
6	7	4	2,835.30	15	4.45	4.18	0
9	10	2	2,959.64	15	4.55	4.73	0
6	2	3	2,126.47	15	4.91	4.82	1
6	40	3	1,417.65	15	4.73	4.77	1
9	10	3	1,701.18	15	4.91	3.86	0
12	10	0	1,417.65	15	4.00	4.77	0
12	10	0	1,417.65	15	4.73	4.77	1
12	3	0	1,701.18	15	4.91	4.77	1
6	20	3	1,417.65	15	4.18	3.91	1
9	10	1	2,835.30	15	4.00	4.91	1
9	10	2	1,417.65	15	4.73	3.95	0
6	2	0	26.49	15	3.73	4.05	1
12	5	3	23.08	15	4.00	4.00	1

9	15	5	20.05	15	3.73	4.00	1
6	10	3	41.78	15	3.82	4.27	0
12	16	4	90.93	15	3.55	4.09	0
0	25	2	59.16	15	3.82	4.09	0
9	3	0	43.42	15	3.73	4.05	1
12	9	4	36.93	15	3.91	3.95	1
6	20	2	33.11	15	4.09	4.05	0
9	9	3	30.22	15	3.64	4.00	1
6	15	2	29.99	15	3.73	4.05	0
12	2	3	19.59	15	3.82	3.95	1
9	25	6	22.68	15	3.73	4.18	1
9	10	1	51.48	15	3.64	4.05	0
6	10	3	27.42	15	3.64	4.18	0
6	3	6	59.69	15	3.73	3.95	1
6	3	0	141.02	15	3.55	4.05	1
6	35	2	36.56	15	3.64	4.05	0
6	12	0	28.35	15	3.73	3.95	0
12	15	3	56.24	15	3.82	4.05	0
6	20	1	45.47	15	3.64	3.91	0
12	20	5	36.56	15	3.82	4.00	0
6	3	0	43.65	15	3.82	4.14	0
9	5	4	68.20	15	3.82	4.14	0
6	30	4	59.69	15	3.64	4.23	0
12	3	0	59.16	15	3.82	3.95	0
12	6	2	70.51	15	3.55	3.91	0
9	12	2	76.94	15	3.91	4.23	0
12	9	2	45.47	15	3.73	4.05	0
6	30	2	78.34	15	3.82	4.09	1
12	20	4	90.04	23	3.82	2.68	0
12	20	4	83.33	30	3.64	2.86	0
9	20	4	106.63	30	3.82	2.73	0
9	23	1	83.24	23	4.09	2.86	1
8	2	4	74.48	40	4.36	3.05	0
9	17	3	76.77	30	4.18	3.09	1
9	27	4	124.64	30	4.55	2.73	1
12	30	2	97.06	28	4.09	2.82	0
6	6	2	125.68	23	4.18	2.50	0
12	19	3	92.99	30	4.64	2.95	0
12	10	1	84.04	30	4.36	2.64	1
12	12	3	62.12	30	4.27	2.86	0
12	20	3	63.18	28	4.36	2.91	1
9	21	4	63.64	28	4.55	2.91	0
9	15	3	52.91	30	4.09	2.73	0
12	21	2	70.97	30	4.27	2.82	1
12	6	2	469.04	40	4.09	2.91	0
12	8	2	1,368.47	40	4.09	2.91	0
9	2	3	81.19	23	4.36	2.68	0

12	30	3	26.66	12	4.45	2.82	0
12	40	1	169.71	28	4.45	2.86	0
12	10	5	136.85	40	4.45	2.95	0
9	6	3	84.04	30	4.36	2.86	0
6	25	2	124.13	30	4.27	2.86	0
12	10	2	150.07	15	4.55	2.82	0
9	18	2	116.24	12	4.36	2.82	0
9	20	5	42.12	40	4.36	2.86	0
12	1	6	102.56	30	4.27	2.73	0
9	18	4	199.42	30	4.09	2.77	0
12	21	5	120.06	30	4.18	3.05	0
6	30	3	49.65	40	4.18	2.91	0
9	15	2	63.03	30	4.00	2.77	0
12	26	3	124.13	30	4.36	2.64	0
9	15	3	63.03	30	4.55	2.68	0
12	11	3	103.53	40	4.45	2.82	0
12	11	2	167.51	15	4.36	2.77	0
12	1	2	116.24	30	4.45	2.77	0
9	22	3	76.92	15	4.27	2.77	0
12	20	2	124.23	28	4.27	2.86	0
12	25	3	70.97	30	4.27	3.00	0
12	11	3	136.85	40	4.09	2.82	0
12	10	3	110.99	15	4.36	2.82	0
12	15	3	90.04	28	4.27	3.00	0
9	15	2	62.12	30	4.27	3.14	0
12	9	4	133.29	30	3.91	3.05	0
12	5	2	62.12	30	4.36	2.95	0
9	10	2	106.45	28	4.18	2.82	0
9	30	3	42.12	28	4.27	2.77	0
9	20	3	81.37	40	4.18	2.86	0
12	13	2	120.06	40	4.27	2.91	0
12	15	2	70.97	30	4.45	2.91	0
12	1	3	95.23	20	4.45	2.82	0
12	5	2	90.04	30	4.36	2.82	0
12	6	3	95.46	30	4.45	2.82	0
12	9	2	102.50	20	4.36	2.77	0
12	13	3	62.12	30	4.18	2.86	0
12	11	3	67.66	30	4.09	2.95	0
12	13	4	136.85	28	4.18	2.95	0
12	10	2	63.49	28	4.27	2.82	0
12	5	1	108.26	30	4.64	2.82	0
9	20	7	83.69	40	3.64	2.68	0
6	30	4	41.84	40	3.45	2.86	0
12	30	1	83.69	40	3.64	2.73	0
9	20	3	21.90	40	3.73	2.86	0
9	46	1	85.47	40	3.73	3.05	0
12	25	2	166.35	40	3.91	3.09	0

6	30	3	133.90	40	3.55	2.73	0
9	10	3	72.82	40	3.91	2.82	0
9	25	4	36.22	40	3.55	2.50	0
9	25	5	56.09	40	3.73	2.95	0
9	20	3	37.98	40	3.91	2.64	0
6	15	3	92.75	40	3.55	2.86	0
9	10	3	206.49	40	3.55	2.91	0
6	30	4	52.39	40	4.18	2.91	0
9	20	3	32.70	40	3.73	2.73	0
12	30	2	27.50	40	3.82	2.82	0
6	15	3	33.38	40	4.00	2.91	0
9	10	3	30.64	40	3.55	2.91	0
12	20	4	74.36	40	4.09	2.68	0
9	20	3	74.36	40	3.73	2.82	0
12	20	3	54.47	40	4.00	2.86	0
12	20	2	46.48	40	3.73	2.95	0
12	25	2	64.63	40	3.82	2.86	0
6	20	3	130.14	40	4.09	2.86	0
9	30	4	46.48	40	3.73	2.82	0
9	10	3	43.49	40	3.73	2.82	0
6	15	3	192.13	40	4.18	2.86	0
9	20	3	45.12	40	3.73	2.73	0
9	20	4	133.90	40	3.91	2.77	0
6	25	2	46.48	40	4.09	3.05	0
12	15	4	172.92	40	3.45	2.91	0
9	27	4	259.37	40	3.82	2.77	0
12	15	4	172.92	40	3.73	2.64	0
6	10	4	86.46	40	3.91	2.68	0
6	35	5	86.46	40	4.18	2.82	0
12	22	5	259.37	40	3.55	2.77	0
9	20	3	172.92	40	3.91	2.77	0
6	25	4	129.69	40	3.73	2.77	0
6	20	3	86.46	40	3.45	2.86	0
9	15	3	86.46	40	3.64	3.00	0
9	17	4	69.17	40	3.91	2.82	0
6	20	3	69.17	40	4.00	2.82	0
9	15	3	86.46	40	3.91	3.00	0
9	23	4	25.94	40	3.91	3.14	0
6	20	4	43.23	40	4.00	3.05	0
12	17	3	86.46	40	3.82	2.95	0
9	20	4	60.52	40	4.00	2.82	0
12	30	5	69.17	40	3.55	2.77	0
6	20	4	34.58	40	3.73	2.86	0
9	17	4	17.29	40	4.09	2.91	0
9	18	4	86.46	40	4.09	2.91	0
12	17	5	77.81	40	3.64	2.82	0
6	23	5	60.52	40	3.91	2.82	0

9	21	4	34.58	40	3.82	2.82	0
9	16	4	43.23	40	3.36	2.77	0
12	14	3	60.52	40	4.00	2.86	0
9	19	4	69.17	40	4.00	2.95	0
9	21	5	60.52	40	3.64	2.95	0
6	15	3	86.46	40	3.91	2.82	0
9	15	2	43.23	40	3.64	2.82	0
6	20	5	159.49	15	3.64	3.05	0
12	18	5	253.68	15	3.64	2.77	0
6	10	5	182.57	15	3.36	3.09	0
12	8	3	176.27	15	3.18	3.14	0
9	15	3	279.80	15	3.55	3.14	0
9	25	1	50.36	15	3.09	3.14	0
9	25	4	11.89	15	3.36	3.14	0
9	10	1	46.17	15	3.36	3.23	0
6	15	3	61.56	15	3.91	3.09	0
12	20	3	142.70	15	4.09	3.14	0
12	10	2	106.32	15	3.64	3.14	0
9	2	5	98.86	15	3.36	3.09	0
9	20	4	159.49	15	3.82	3.27	0
9	12	3	125.91	15	3.91	3.09	0
12	20	4	12.59	15	3.45	2.86	0
9	8	3	159.49	15	3.55	3.14	0
6	20	6	73.87	15	3.82	3.05	0
6	30	4	11.89	15	3.36	3.14	0
9	17	3	92.33	15	3.18	3.27	0
12	15	3	106.32	15	3.64	3.14	0
12	20	3	190.26	15	3.55	3.18	0
12	8	2	25.18	15	3.36	3.09	0
12	10	3	176.27	15	3.91	3.14	0
9	13	4	126.84	15	3.55	3.14	0
12	20	4	23.78	15	3.36	3.05	0
12	15	3	11.89	15	3.18	3.14	0
6	21	5	12.59	15	3.82	3.27	0
9	15	2	161.58	15	3.64	3.09	0
6	20	5	117.52	15	3.27	3.14	0
12	20	3	106.32	15	3.55	3.23	0
6	18	4	193.06	15	3.45	3.50	0
6	20	3	160.88	15	3.36	3.45	0
12	8	3	128.71	15	3.91	3.36	0
12	20	4	160.88	15	3.45	3.36	0
9	15	3	139.43	15	3.36	3.36	0
12	20	4	160.88	15	3.91	3.36	0
9	20	4	128.71	15	3.45	3.36	0
9	30	3	160.88	15	3.36	3.36	0
12	10	2	160.88	15	3.91	3.36	0
6	20	5	128.71	15	3.45	3.36	0

9	20	4	160.88	15	3.36	3.36	0
9	40	2	53.63	15	3.91	3.36	0
6	25	3	160.88	15	3.45	3.36	0
12	30	4	139.43	15	3.36	3.36	0
6	30	4	268.14	15	3.91	3.36	0
12	10	3	128.71	15	3.45	3.36	0
12	12	4	117.98	15	3.36	3.36	0
6	17	4	128.71	15	3.91	3.36	0
12	18	4	160.88	15	3.45	3.36	0
9	20	4	128.71	15	3.36	3.36	0
12	30	3	160.88	15	3.91	3.36	0
9	15	2	214.51	15	3.45	3.36	0
6	28	3	268.14	15	3.36	3.36	0
12	35	4	21.45	15	3.91	3.36	0
6	20	2	21.45	3.5	3.45	3.36	0
12	20	2	128.71	15	3.36	3.36	0
12	20	4	160.88	15	3.91	3.36	0
6	40	3	214.51	15	3.45	3.36	0
12	14	4	107.26	15	3.64	3.36	0
12	15	2	53.63	3.5	3.91	3.36	0

Access to credit	Group membership
0.00	1
0.00	1
0.00	0
0.00	1
0.00	0
0.00	1
0.00	0
0.00	1
0.00	0
0.00	0
0.00	1
0.00	0
0.00	0
0.00	1
1.00	1
1.00	0
1.00	1
1.00	1
1.00	1
1.00	0
1.00	1
1.00	0
0.00	0
0.00	0
0.00	0
0.00	0
0.00	0
1.00	0
0.00	0
1.00	1
0.00	0
0.00	1
0.00	0
0.00	0
0.00	1
0.00	0
0.00	0
0.00	0
0.00	0
0.00	0
0.00	0
0.00	0
0.00	0
0.00	0
0.00	0

0.00	0
0.00	0
0.00	0
0.00	0
0.00	0
0.00	0
0.00	0
0.00	0
0.00	0
0.00	0
0.00	0
0.00	0
0.00	0
0.00	0
0.00	0
1.00	1
1.00	1
1.00	1
1.00	1
1.00	1
1.00	1
1.00	1
1.00	1
1.00	1
1.00	1
1.00	1
1.00	1
1.00	1
1.00	1
1.00	1
1.00	1
1.00	1
1.00	1
1.00	1
1.00	1
1.00	1
1.00	1
1.00	1
1.00	1
1.00	1
1.00	1
1.00	1
1.00	1
1.00	1
1.00	1
0.00	1
0.00	0

0.00	1
0.00	0
0.00	0
0.00	0
0.00	0
0.00	1
0.00	1
0.00	0
0.00	1
0.00	1
0.00	0
0.00	0
0.00	0
0.00	0
0.00	0
0.00	0
0.00	0
0.00	0
0.00	0
0.00	1
0.00	0
0.00	0
0.00	0
0.00	0
0.00	0
0.00	0
0.00	0
0.00	0
0.00	0
0.00	0
0.00	0
0.00	0
0.00	0
0.00	0
0.00	0
0.00	1
0.00	0
0.00	0
0.00	0
0.00	0
0.00	0
0.00	0
0.00	1
0.00	0
1.00	0
1.00	0
0.00	1

0.00	0
0.00	0
0.00	0
0.00	0
0.00	0
0.00	0
0.00	0
0.00	0
0.00	0
0.00	0
0.00	0
0.00	1
0.00	0
0.00	0
0.00	0
0.00	0
0.00	0
0.00	0
0.00	1
0.00	0
0.00	0
0.00	0
0.00	0
0.00	0
0.00	0
0.00	0
0.00	0
0.00	0
0.00	0
0.00	0
0.00	0
0.00	0
0.00	0
0.00	0
0.00	0
0.00	0
0.00	1
0.00	0
0.00	0
0.00	0
0.00	0
0.00	0
0.00	0
0.00	0
0.00	0
0.00	0
0.00	0

0.00	0
0.00	0
0.00	0
0.00	0
0.00	0
0.00	0
0.00	0
0.00	0
0.00	0
0.00	0
0.00	0
0.00	0
0.00	0
0.00	0
0.00	0
0.00	0
0.00	0
0.00	0
0.00	0
0.00	0
0.00	0
0.00	0
0.00	0
0.00	0
0.00	0
0.00	0
0.00	0
0.00	0
0.00	0
0.00	0
0.00	0
0.00	0
0.00	0
0.00	0
0.00	0
0.00	0
0.00	0
0.00	0
0.00	0
0.00	0
0.00	0
0.00	0
0.00	0
0.00	0
0.00	0
0.00	0
0.00	0

0.00	0
0.00	0
0.00	0
0.00	0
0.00	0
0.00	0
0.00	0
0.00	1
0.00	1
0.00	0
0.00	0
0.00	0
0.00	0
0.00	0
0.00	0
0.00	0
0.00	0
0.00	0
0.00	0
0.00	0
0.00	0
0.00	0
0.00	0
0.00	0
0.00	0
0.00	0
0.00	0
0.00	0
0.00	0
0.00	0
0.00	0
0.00	0
0.00	0
0.00	0
0.00	0
0.00	0
0.00	0
0.00	0
0.00	0
0.00	0
0.00	0
0.00	0
0.00	0
0.00	0
0.00	0
0.00	0
0.00	0

0.00	0
0.00	0
0.00	0
0.00	0
0.00	0
0.00	0
0.00	0
0.00	0
0.00	0
0.00	0
0.00	0
0.00	0
0.00	0
0.00	0
0.00	0
0.00	0
0.00	0
0.00	0
0.00	0
0.00	0
